# A Coupled Mathematical Model of the Intracellular Replication of Dengue Virus and the Host Cell Immune Response to Infection

**DOI:** 10.3389/fmicb.2020.00725

**Published:** 2020-04-29

**Authors:** Carolin Zitzmann, Bianca Schmid, Alessia Ruggieri, Alan S. Perelson, Marco Binder, Ralf Bartenschlager, Lars Kaderali

**Affiliations:** ^1^Center for Functional Genomics of Microbes, Institute of Bioinformatics, University Medicine Greifswald, Greifswald, Germany; ^2^Theoretical Biology and Biophysics, Los Alamos National Laboratory, Los Alamos, NM, United States; ^3^Department of Infectious Diseases, Molecular Virology, Heidelberg University, Heidelberg, Germany; ^4^Research Group “Dynamics of Early Viral Infection and the Innate Antiviral Response”, Division Virus-Associated Carcinogenesis (F170), German Cancer Research Center (DKFZ), Heidelberg, Germany

**Keywords:** dengue virus, mathematical model, innate immune response, virus replication, computational simulation

## Abstract

Dengue virus (DV) is a positive-strand RNA virus of the *Flavivirus* genus. It is one of the most prevalent mosquito-borne viruses, infecting globally 390 million individuals per year. The clinical spectrum of DV infection ranges from an asymptomatic course to severe complications such as dengue hemorrhagic fever (DHF) and dengue shock syndrome (DSS), the latter because of severe plasma leakage. Given that the outcome of infection is likely determined by the kinetics of viral replication and the antiviral host cell immune response (HIR) it is of importance to understand the interaction between these two parameters. In this study, we use mathematical modeling to characterize and understand the complex interplay between intracellular DV replication and the host cells' defense mechanisms. We first measured viral RNA, viral protein, and virus particle production in Huh7 cells, which exhibit a notoriously weak intrinsic antiviral response. Based on these measurements, we developed a detailed intracellular DV replication model. We then measured replication in IFN competent A549 cells and used this data to couple the replication model with a model describing IFN activation and production of IFN stimulated genes (ISGs), as well as their interplay with DV replication. By comparing the cell line specific DV replication, we found that host factors involved in replication complex formation and virus particle production are crucial for replication efficiency. Regarding possible modes of action of the HIR, our model fits suggest that the HIR mainly affects DV RNA translation initiation, cytosolic DV RNA degradation, and naïve cell infection. We further analyzed the potential of direct acting antiviral drugs targeting different processes of the DV lifecycle *in silico* and found that targeting RNA synthesis and virus assembly and release are the most promising anti-DV drug targets.

## Introduction

Dengue virus (DV) is the most prevalent vector-borne virus in the world, with an estimated global number of 390 million new infections annually, thereof 90 million with clinical manifestations, including severe dengue disease (Bhatt et al., 2013). DV poses a huge burden on human populations and health systems in affected countries, and significantly impacts the economy in tropical countries, including the southern United States (WHO, 2012). Fueled by climate change and globalization and accelerated further by viral evolution, the expansion of DV is expected to increase further (Murray et al., 2013). DV is transmitted mainly by female *Aedes* mosquitos, and with the spread of its vector, DV is spreading as well (Campbell et al., 2015). In consequence, the global incidence of DV infection has already risen 30-fold during the past 50 years. Infection with DV causes flu-like symptoms but is occasionally associated with severe complications. The fatality rate of dengue infection is between 1 and 5%, and below 1% with proper symptomatic treatment (Ranjit and Kissoon, 2011). There is no antiviral therapy available against DV, and the recently approved vaccine has limited efficacy and depends on baseline serostatus of the vaccine recipient (World Health Organization, 2016).

DV infects dendritic cells (DC), B cells, T cells, monocytes, macrophages, but also the liver. DV is an enveloped, positive-sense (+)RNA virus of the *Flaviviridae* family within the genus *Flavivirus*, consisting of four distinct serotypes (DV1/2/3/4). Its approximately 10.7 kb genome encodes the three structural proteins capsid (C), precursor membrane (prM), and envelope (E) protein and seven non-structural proteins (NS1, NS2A, NS2B, NS3, NS4A, NS4B, and NS5). Upon entry into the host cell, the viral RNA genome is translated at the rough endoplasmic reticulum (rER) giving rise to a polyprotein, ~3,400 amino acids in length, which is co- and post-translationally cleaved by viral and host proteases into the structural and non-structural proteins (Neufeldt et al., 2018). DV induces membrane alterations at the rER, forming membrane invaginations. The viral RNA genome is amplified in these replication compartments (RC), starting with minus-strand synthesis to obtain a double-stranded RNA (dsRNA) intermediate which then serves as template for further plus-strand synthesis. The newly synthesized viral (+)RNA genomes leave the RC and are then either packaged into virions, which after maturation are released from the infected cell, or are again used for the next round of viral RNA translation (Bartenschlager and Miller, 2008; Rodenhuis-Zybert et al., 2010; Tuiskunen Bäck and Lundkvist, 2013; Screaton et al., 2015).

The host cell's defense against DV is mediated via pattern recognition receptors (PRRs), in case of DV mainly via the endosomal Toll-like receptors (TLR3/TLR7/TLR8) and the cytosolic RNA helicases *retinoic acid inducible gene I* (RIG-I) and *melanoma differentiation associated gene 5* (MDA-5) (Muñoz-Jordán and Fredericksen, 2010; Morrison et al., 2012). TLR3 recognizes dsRNA, while TLR7 and TLR8 recognize viral single-stranded RNA (Xagorari and Chlichlia, 2008). All three TLRs activate signaling cascades that lead to the production of interferon α/β (IFN α/β) and inflammatory cytokines. RIG-I/MDA-5 signals via *mitochondrial antiviral signaling protein* (MAVS) and *tumor necrosis factor receptor-associated factor 3* (TRAF3), activating *tank-binding kinase 1* (TBK1) and ultimately phosphorylating *interferon regulatory factor 3* (IRF3) and activating *nuclear factor kappa B* (NF-κB). The subsequent type I (α/β) and type III (λ) IFN production induces the activation of hundreds of IFN stimulated genes (ISGs), bringing the cells into an antiviral state and resulting in an inhibition of DV (Nasirudeen et al., 2011; Tuiskunen Bäck and Lundkvist, 2013; Dalrymple et al., 2015).

DV, however, is not defenseless, and has evolved a number of mechanisms antagonizing the antiviral response of the cell both at the level of activation of the host cell immune response (HIR) and the induced effector phase. For instance, 2'-O-methylation of the DV RNA genome, mediated by NS5, was shown to slow down the activation kinetics of the IFN response (Schmid et al., 2015). In addition, the DV NS2B-3 protease cleaves the stimulator of interferon genes (STING), thus reducing type I IFN production (Diamond and Pierson, 2015). In fact, several groups have shown that the suppression of the early IFN induction by DV is critical for successful virus infection and replication (Shresta et al., 2004; Perry et al., 2009). Moreover, Schmid et al. (2015) have shown that the ability of IFN to control DV spread might be stochastic and “leaky.” While secreted IFN protects surrounding naïve cells from infection, this protection is incomplete with cells infected with DV prior to activation of the IFN response (Schmid et al., 2015). DV replication occurs inside membrane vesicles corresponding to invaginations into the rER lumen, likely shielding viral dsRNA intermediates from recognition by the HIR (Welsch et al., 2009). At the level of the effector phase, DV NS5, which contains the enzymatic activity for capping and amplification of the viral RNA genome, was shown to bind to and induce the degradation of the signal transducer and activator of transcription factor (STAT) 2 via a proteasome-dependent mechanism (reviewed in Neufeldt et al., 2018), thus blocking ISG induction downstream of the IFN receptor. Therefore, the interplay between DV and the innate immune response (IIR) is complex, and its exact magnitude and dynamics likely impact and possibly determine clinical outcome of the infection.

Mathematical modeling is a valuable tool to study complex dynamical systems and has been employed to analyze infection dynamics for a number of different viruses (Zitzmann and Kaderali, 2018). Most previous work on modeling viral infection has built on the basic model introduced by Nowak and Bangham (1996) and Nowak et al. (1996), focusing on the dynamics of susceptible cells, infected cells, and virus at the cell population level. Especially, the within-host dynamics of human immunodeficiency virus (HIV) and hepatitis C virus (HCV) have been studied in detail with simple models on the cell population scale with regard to the antiviral immune response and treatment opportunities (Ho et al., 1995; Wei et al., 1995; Perelson et al., 1996, 1997; Bonhoeffer et al., 1997; Neumann, 1998; Stafford et al., 2000; Perelson, 2002; Rong and Perelson, 2009; Perelson and Ribeiro, 2013; Perelson and Guedj, 2015). In the case of DV modeling, these so-called target cell-limited models have been linked to the adaptive immune response via modeling of antibodies and T cell responses (Clapham et al., 2014; Ben-Shachar et al., 2016), and the IIR by IFN (Ben-Shachar and Koelle, 2014; Schmid et al., 2015). Several authors have furthermore developed intracellular replication models for related viruses (Dahari et al., 2007; Heldt et al., 2012, 2013; Binder et al., 2013; Guedj et al., 2013; Clausznitzer et al., 2015; Laske et al., 2016; Benzine et al., 2017; Aunins et al., 2018), but to our knowledge there is no mathematical model describing the intracellular steps of DV replication to date. In this manuscript, we focus on the highly dynamic initial phase post cell infection and developed a detailed differential equations model capable of quantitatively describing the intracellular infection dynamics. We measured viral replication in two different cell lines, Huh7 cells (with very little HIR) and A549 cells (with high HIR competence). By integrating the main steps of the HIR into the model, we were able to describe the infection kinetics in both cell types. Our investigation focuses on the cell line-specific impact of host factors, which determine RNA synthesis efficiency, and the involvement of the HIR. Using our mathematical model, we identified possible antiviral modes of action of the HIR on the DV lifecycle and the viral countermeasures suppressing DV recognition and activation of the HIR. We further identified the most sensitive processes in the DV lifecycle, which might constitute promising antiviral drug targets, and we evaluated possible antiviral intervention strategies *in silico*.

## Materials and Methods

### Measuring DV Replication in Huh7 and A549 Cells

#### Cell Lines

A549 and Huh7 cells were cultivated at 37°C, 5% CO^2^ in of DMEMcplt (2 mmol/L L-glutamine, non-essential amino acids, 100 U/ml penicillin, 100 μg/ml streptomycin and 10% fetal calf serum).

#### Kinetics Experiments (120830 and 120921)

2 × 10^5^ A549 and Huh7 cells were seeded 1 day prior infection. Cells were infected with DV reporter virus expressing Renilla Luciferase (Schmid et al., 2015) at a MOI of 10. After 1 h, the inoculum was removed and cells washed thrice with sterile PBS prior addition of DMEMcplt. Cells were incubated at 37°C for the indicated time points.

#### Infectivity Titers

Supernatants were harvested and filtered through a 0.45 μm-pore size membrane. Supernatants were supplemented with 15 mM HEPES and stored at −80°C. Infectivity titers of virus supernatants were determined by limiting dilution assay using Huh7.5 cells as described elsewhere (Lindenbach et al., 2005).

#### Interferon Lambda ELISA

Supernatants of infected cells were supplemented with 1% (v/v) Triton X-100 to inactivate DENV infectious particles and subsequently stored at −80°C until further use. Interferon lambda protein release was determined by VeriKine-DIYTM Human Interferon Lambda/IL-28B/29/28A ELISA (PBL Interferon Source, USA) with an assay range of 62.5 to 4,000 pg/ml. ELISA procedure was conducted according to the manufacturer's protocol using high binding 96 well ELISA microplates (Greiner Bio-One, Frickenhausen, Germany). The optical density of each well was determined immediately using a microplate reader set to 450 nm.

#### RT-qPCR

Cells were lysed for RNA extraction and subsequent qRT-PCR analysis by adding 350 μl RA1 lysis buffer (Macherey-Nagel, Düren, Germany) supplemented with 1% β-mercapto-ethanol and stored at −80°C. Total RNA was extracted using the NucleoSpin RNA II Kit (Macherey-Nagel) as recommended by the manufacturer. RT-qPCR was described elsewhere (Schmid et al., 2015). The following primers were used: DENV2 (forward 5′-GCC CTT CTG TTC ACA CCA TT-3′; reverse 5′-CCA CAT TTG GGC GTA AGA CT-3′); IFIT1 (forward 5′- GAA GCA GGC AAT CAC AGA AA-3′; reverse 5′-TGA AAC CGA CCA TAG TGG AA-3′). GAPDH mRNA (primer forward 5′ - GAA GGT GAA GGT CGG AGT C−3′; reverse 5′ - GAA GAT GGT GAT GGG ATT TC – 3′) was used for normalization of input RNA.

#### Luciferase Assay

Cells were lysed in 100 μl Luciferase lysis buffer (1% Triton X-100, 25 mM glycyl-glycine, 15 mM MgSO_4_, 4 mM EGTA, and 1 mM DTT, pH 7.8) and stored at −80°C. For detection of Renilla luciferase activity, 20 μl lysate was mixed with 100 μl assay buffer (25 mM glycyl-glycine, 15 mM MgSO_4_, 4 mM EGTA, and 15 mM potassium phosphate pH 7.8) containing 1.4 μM coelenterazine (P.J.K). All measurements were done in duplicate by using a tube luminometer (Berthold, Pforzheim, Germany). Replication efficiency was determined by normalization to the 2 h values reflecting infection efficiency.

### Mathematical Model

**Figure 1:**
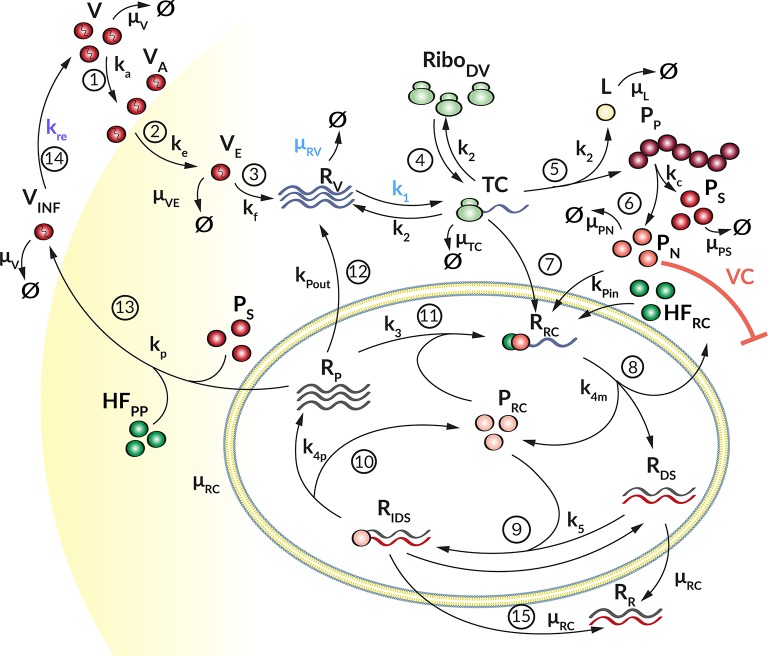
Graphical illustration of the DV replication model. (1) The Virus (*V*) attaches to a permissive cell (*k*_*a*_) and (2) enters via receptor-mediated endocytosis (*k*_*e*_). (3) The virus in the endosome (*V*_*E*_) is degraded with rate (μ_*V*_*E*__). The viral and endosomal membrane fuse (*k*_*f*_) and release the viral RNA genome (*R*_*V*_), which is degraded with rate μ_*R*_*V*__. (4) Ribosomes (*Ribo*_*DV*_) bind (*k*_1_) at the viral RNA genome, forming a translation complex (*TC*), which in turn is degraded with rate μ_*TC*_. (5) The viral genome is translated (*k*_2_) into a long polyprotein (*P*_*P*_) and (6) subsequently cleaved (*k*_*c*_) into structural (*P*_*S*_) and non-structural proteins (*P*_*N*_), which degrade with rate μ_*P*_*S*__ and μ_*P*_*N*__, respectively. During the translation, luciferase (*L*) is produced as a marker for translation activity and is degraded with rate μ_*L*_. (7) The TC together with non-structural proteins and host factors (HF) initiating the formation (*k*_*Pin*_) of a replication complex (*R*_*RC*_). (8) The antisense synthesis (*k*_4*m*_) leads to production of double-stranded RNA (dsRNA) that (9) binds to non-structural proteins in the Replication Compartment RC (*P*_*RC*_) forming (*k*_5_) a minus-strand RNA intermediate complex (*R*_*IDS*_). *R*_*IDS*_ in turn (10) initiates plus-strand RNA synthesis (*k*_4*p*_). (11) The newly synthesized plus-strand RNA (*R*_*P*_) can be exported out of the RC into the cellular cytoplasm (*k*_*Pout*_), (12) undergoes another round of replication (*k*_3_) or (13) is packed into virus particles and released (*k*_*r*_) from the host cell (14), where it can infect naïve cells (*k*_*re*_) for a further round of replication. Species in the RC degrade with constant rate μ_*RC*_, (15) except the dsRNA species which get transported out the RC with rate constant μ_*RC*_ resulting in an accumulation of dsRNA in the cytoplasm (*R*_*R*_). Extracellular IFN and ISG proteins have an antiviral effect (AE**⊣** see Figure 2) on the DV lifecycle via different mechanisms (*k*_1_, *k*_*re*_, and μ_*R*_*V*__) while the *P*_*N*_ dependent countermeasures (VC**⊣**) targeting HIR pathways (*k*_*rig*_ and *k*_*jak*_, see Figure 2).

**Figure 2:**
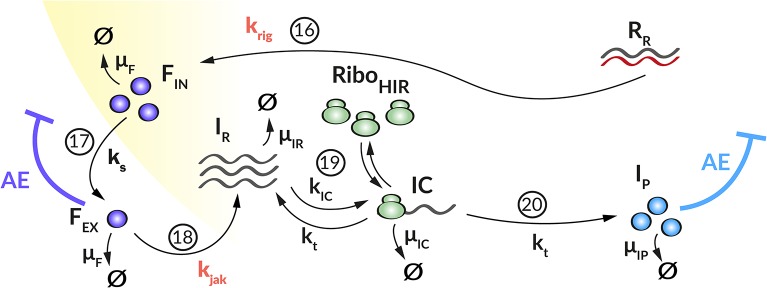
Graphical illustration of the HIR sub-model. (16) Upon recognition of cytoplasmic dsRNA (*R*_*R*_) via the RIG-I pathway (*k*_*rig*_), IFN (*F*_*IN*_) is produced that (17) is released from the cell (*k*_*s*_). (18) Extracellular IFN (*F*_*EX*_) activates the JAK/STAT pathway (*k*_*jak*_) that leads to the production of ISG mRNA (*I*_*R*_). (19) Ribosomes (*Ribo*_*HIR*_) bind to ISG mRNA in order to form (*k*_*IC*_) a translation initiation complex (*IC*) that (20) translates (*k*_*t*_) the ISG mRNA into ISG proteins (*I*_*P*_). Extracellular IFN and ISG proteins have an antiviral effect (AE**⊣**) on the DV lifecycle via different mechanisms (*k*_1_, *k*_*re*_, and μ_*R*_*V*__, see Figure 1) while the *P*_*N*_ dependent countermeasures (VC**⊣**, see Figure 1) targeting HIR pathways (*k*_*rig*_ and *k*_*jak*_).

We developed a mechanistic mathematical model using ordinary differential equations (ODEs) and mass action kinetics to analyze the interplay between DV and the HIR. We have previously published a detailed intracellular replication model for HCV (Binder et al., 2013). Both DV and HCV are closely related (+)RNA viruses of the same family, and their intracellular replication proceeds in similar steps. We therefore used the mathematical model of HCV replication as the basis for the model structure of the current DV model. This model was then extended to the full virus lifecycle by adding infection and assembly of new virus particles to the model. We then adapted the model to DV where necessary (see details below), refitted model parameters on the Huh7/DV infection data and complemented the replication model by an HIR sub-model that comprises the key components of the IIR. Figure 1 gives an overview of the DV replication sub-model and Figure 2 shows the HIR sub-model; we describe each of the main model components in the following.

#### DV Replication

Our DV replication sub-model (Figure 1) is composed of four main processes in the DV lifecycle: (1) Binding of DV particles to the cell surface and viral entry via endocytosis. (2) Uncoating and release of the viral RNA genome into the cytoplasm, followed by translation into a polyprotein that is subsequently cleaved into the structural and non-structural viral proteins. (3) The non-structural viral proteins initiate the formation of a RC, in which the viral RNA replication takes place via a dsRNA intermediate. (4) The newly synthesized (+)RNA can then be used as a template for further RNA replication, for protein production at the ribosomes, or it is used together with the structural proteins to assemble new virus particles, which are then released from the cell. The cycle thereafter starts over again with further infection of naïve cells.

We model the infection process by the following three ODEs, where *V* describes extracellular virus, *V*_*A*_ is virus that has attached to the host cell but is still at the cell surface, and *V*_*E*_ is virus that has been endocytosed:

(1) $$\begin{array}{l} \frac{dV}{dt}=\;k_{re}V_{INF}-k_{a}V-\mu_{V}V-wV \end{array}$$

(2) $$\begin{array}{l} \frac{dV_{A}}{dt}=k_{a}V-k_{e}V_{A} \end{array}$$

(3) $$\begin{array}{l} \frac{dV_{E}}{dt}=k_{e}V_{A}-k_{f}V_{E}-\mu_{V_{E}}V_{E} \end{array}$$

Equation (1) and (2) describe how extracellular virus (*V*) binds at the cell surface of a permissive host cell with rate constant *k*_*a*_ and degrades with rate constant μ_*V*_ (Equation 1). To keep the model simple, we assume that attachment is non-reversible. According to the experimental set-up, the cells were washed to remove unbound virus from the initial infection. This is considered in the model through the term *wV*, where *w* is modeled as

(4) $$\begin{array}{l} w=\omega_{s}\frac{1}{\sqrt{2\pi \omega_{d}^{2}}}e^{-\;\frac{{\left(t-\omega_{t}\right)}^{2}}{2\omega_{d}^{2}}}, \end{array}$$

with washing time point ω_*t*_, washing duration ω_*d*_, and washing strength ω_*s*_ (for more details, see Supplementary Material). The term *k*_*re*_*V*_*INF*_ in Equation (1) describes newly released infectious virus particles from previous rounds of virus replication, which can infect naïve cells. Equations (2) and (3) describe cell entry: Attached virus (*V*_*A*_) enters the cell with rate constant *k*_*e*_ via receptor-mediated endocytosis. Subsequently, virus in the endosome (*V*_*E*_) undergoes conformational changes of the nucleocapsid, leading to fusion of the viral and endosomal membranes with rate constant *k*_*f*_ (Equation 3). Endocytosed virus *V*_*E*_ decays with rate constant μ_*V*_*E*__.

The steps associated with RNA translation are described by the following ODEs, which are based on our previously published HCV replication model (Binder et al., 2013). Here, *R*_*V*_ describes viral (+)RNA in the cytoplasm, *TC* corresponds to active translation complexes, *P*_*P*_ describes viral polyprotein, and *P*_*S*_ and *P*_*N*_ are representatives for the structural and non-structural viral proteins, respectively. In our experimental data, *P*_*P*_ is measured via a bicystronic luciferase reporter system, the species *L* is therefore required for model fitting purposes and gives luciferase protein concentration, which degrades with rate constant μ_*L*_. The following ODEs describe the temporal evolution of these species:

(5) $$\begin{array}{l} \frac{dR_{V}}{dt}=k_{f}V_{E}-k_{1}R_{V}\left({Ribo_{DV}}_{tot}-TC\right)+k_{2}TC\\ \;+k_{Pout}R_{P}-\mu_{R_{V}}R_{V} \end{array}$$

(6) $$\begin{array}{l} \frac{dTC}{dt}=k_{1}R_{V}\left({Ribo_{DV}}_{tot}-TC\right)-k_{2}TC\\ \;-\;k_{Pin}P_{N}TC\left(HF_{RC_{0}}-R_{RC}\right)-\mu_{TC}TC \end{array}$$

(7) $$\begin{array}{l} \frac{dP_{P}}{dt}=k_{2}TC-k_{c}P_{P} \end{array}$$

(8) $$\begin{array}{l} \frac{dL}{dt}=\;k_{2}TC-\mu_{L}L \end{array}$$

(9) $$\begin{array}{l} \frac{dP_{S}}{dt}=k_{c}P_{P}-\mu_{P_{S}}P_{s}-N_{P_{S}}\;v_{p} \end{array}$$

(10) $$\begin{array}{l} \frac{dP_{N}}{dt}=k_{c}P_{P}-k_{Pin}P_{N}TC\left(HF_{RC_{0}}-R_{RC}\right)-\mu_{P_{N}}P_{N} \end{array}$$

After cell entry, the viral RNA genome *R*_*V*_ is released into the cytoplasm and is subsequently translated giving rise to viral protein or is degraded with rate constant μ_*R*_*V*__ (Equation 5). Free ribosomes (*Ribo*_*DV*_) reversibly bind to the viral RNA genome (*R*_*V*_) to form a translation initiation complex (*TC*, Equation 6) with rate constant *k*_1_. We assume that the total number of ribosomes, *Ribo*_*D*_*V*__*tot*__, is constant, hence free ribosomes available for translation are given by *Ribo*_*DV*_(*t*) = *Rib*_*o*_*DV*_0_ − *TC* and it is not necessary to introduce a separate equation for the ribosomes. *HF*_*R*_*C*__0__ represents one or more unspecified host cell factor(s) that are required for formation of the RC and is assumed constant (see Table S1). Upon translation of the viral genome into a polyprotein (*P*_*P*_, Equation 7) with rate constant *k*_2_, the translation initiation complex (*TC*) dissociates into free viral RNA (*R*_*V*_) and ribosomes (*Ribo*_*DV*_). Furthermore, during *TC* degradation with rate constant μ_*TC*_, ribosomes dissociate from the complex: *Ribo*_*DV*_ + *R*_*V*_ → *TC* → *Ribo*_*DV*_ + *R*_*V*_ + *P*_*P*_ + *L*. We measure polyprotein production using a luciferase reporter system, and hence include luciferase (*L*, Equation 8) into the mathematical model. *L* is produced with rate constant *k*_2_ and is degraded with rate constant μ_*L*_. The polyprotein (*P*_*P*_) is cleaved into structural (*P*_*S*_) and non-structural proteins (*P*_*N*_) with rate constant *k*_*c*_, which degrade with rate constants μ_*P*_*S*__ and μ_*P*_*N*__, respectively. We note here further that later in the virus lifecycle, the structural proteins (*P*_*S*_, Equation 9) are packed together with newly synthesized (+)RNA (*R*_*P*_) into virions, thus the corresponding term involving *v*_*p*_ in equation (9), which is detailed in equation (17) below. Furthermore, the non-structural proteins (*P*_*N*_), e.g., the RNA-dependent-RNA polymerase, are required for viral RNA synthesis (compare Equation 11).

Equations (11) to (16) describe the viral RNA replication inside the replication vesicles. The species *R*_*RC*_ describes a replication intermediate complex for minus strand RNA synthesis inside the RC, whereas *R*_*IDS*_ is the corresponding intermediate complex for (+)RNA production. *R*_*DS*_ and *R*_*P*_ describe dsRNA and single (+)RNA in the RC. *P*_*RC*_ corresponds to the viral RNA polymerase in the RC that is required for RNA replication. Replication is thus modeled by the following equations:

(11) $$\begin{array}{l} \frac{dR_{RC}}{dt}=k_{Pin}P_{N}TC\left(HF_{RC_{0}}-R_{RC}\right)-k_{4m}R_{RC}\\ \;+k_{3}R_{P}P_{RC}\left(HF_{RC_{0}}-R_{RC}\right)-\mu_{RC}R_{RC} \end{array}$$

(12) $$\begin{array}{l} \frac{dR_{DS}}{dt}=k_{4m}R_{RC}-k_{5}R_{DS}P_{RC}+k_{4p}R_{IDS}-\mu_{RC}R_{DS} \end{array}$$

(13) $$\begin{array}{l} \frac{dR_{IDS}}{dt}=k_{5}R_{DS}P_{RC}-k_{4p}R_{IDS}-\;\mu_{RC}R_{IDS} \end{array}$$

(14a) $$\begin{array}{l} \frac{dR_{R}}{dt}=\mu_{RC}(R_{DS}+R_{IDS})-\mu_{R_{V}}R_{R} \end{array}$$

(15) $$\begin{array}{l} \frac{dP_{RC}}{dt}=k_{4m}R_{RC}-k_{3}R_{P}P_{RC}\left(HF_{RC_{0}}-R_{RC}\right)\\ \;-k_{5}R_{DS}P_{RC}+k_{4p}R_{IDS}-\mu_{RC}P_{RC} \end{array}$$

(16) $$\begin{array}{l} \frac{dR_{P}}{dt}=\;k_{4p}R_{IDS}-k_{3}R_{P}P_{RC}\left(HF_{RC_{0}}-R_{RC}\right)\\ \;-k_{Pout}R_{P}-v_{p}-\;\mu_{RC}R_{P} \end{array}$$

By analogy to HCV, we assume here that the initiation of RNA replication occurs from freshly translated viral RNA, hence Equations (6) and (11) model RNA import into the RC from *TC* (instead of *R*_*V*_) with rate constant *k*_*Pin*_. At the same time, non-structural proteins (*P*_*N*_), required for RNA synthesis, and an unspecified host factor *HF*_*RC*_, required for the formation of the RC, are imported into the RC. These assumptions directly match those made in our published HCV replication model (Binder et al., 2013). As the total amount of host factor *HF*_*RC*_ is assumed constant during the time scales considered in the model, a separate equation for *HF*_*RC*_ is not necessary. We furthermore note that, while numerous replication vesicles can be observed during DV replication in every single cell (Welsch et al., 2009), we assume in the model that the sum of all replication vesicles is regarded as a single, large replication compartment, compare (Dahari et al., 2007).

After formation of the replication initiation complex *R*_*RC*_, minus-strand RNA synthesis is initiated with rate constant *k*_4*m*_, leading to the production of dsRNA (*R*_*DS*_, Equation 12) and liberation of viral proteins which remain in the RC (*P*_*RC*_, Equation 15). For the synthesis of (+)RNA (*R*_*P*_, Equation 16), dsRNA (*R*_*DS*_) binds again to *P*_*RC*_ with constant rate *k*_5_ in order to form a minus-strand RNA intermediate complex (*R*_*IDS*_, Equation 13). The minus-strand RNA intermediate complex (*R*_*IDS*_) serves as a template for (+)RNA synthesis with constant rate *k*_4*p*_ and subsequently dissociates into dsRNA (*R*_*DS*_) and viral protein (*P*_*RC*_). The newly synthesized (+)RNA (*R*_*P*_) can then either be transported out of the RC into the cytoplasm with rate constant *k*_*Pout*_, it can be used for a further round of replication with rate constant *k*_3_, or it is used to assemble new virions, which are then released from the cell (*v*_*p*_). We assume that all species in the RC are protected from active degradation, and decay together with the membrane vesicles with a common rate constant μ_*RC*_.

Since the RCs might represent a protective environment for DV replication by shielding DV RNA from the host's immune response recognition (Scutigliani and Kikkert, 2017), we introduced a cytosolic dsRNA species (*R*_*R*_, Equation 14a). Therefore, for the dsRNA species within the RC, *R*_*DS*_ and *R*_*IDS*_, μ_*RC*_ represents a transfer rate into the cytoplasm and leads to the accumulation of cytosolic dsRNA that is detectable by the innate immune sensor (Chazal et al., 2018), while the RNA species within the RC remain protected. In order to account for a slow transfer rate without introducing another model parameter, we use the RNA degradation rate inside the replication compartment, μ_*RC*_, for this purpose. Similar to the single stranded RNA species within the cytoplasm, the cytosolic dsRNA degrades with rate μ_*R*_*V*__.

Finally, Equations (17) to (19) model the assembly and release of new virus particles. To produce one infectious virion, the newly synthesized (+)RNA (*R*_*P*_) assembles together with structural proteins: 180 C proteins, 180 E, and 180 prM/M proteins (Kuhn et al., 2002). Moreover, it has been shown that non-structural proteins support DV particle production as well, e.g., DV NS1 (Scaturro et al., 2015). However, we observed during model fitting that non-structural proteins were not rate limiting for virus assembly and therefore we neglected *P*_*N*_ in the assembly process, while we focused on structural proteins and host factors required or participating in virus assembly and release (for more information see Supplementary Material).

We model DV assembly and release (*v*_*p*_) using a Michaelis-Menten type equation, as

(17) $$\begin{array}{l} v_{p}=\;k_{p}R_{P}\underset{j}{\prod }\frac{P_{j}}{K_{D}\cdot N_{P_{j}}\;+P_{j}}, \end{array}$$

with *j* ∈ {*P*_*S*_, *HF*_*PP*_}, *N*_*P*_*j*__ the number of each protein *P*_*j*_, and cell line-specific virion release rate constant *k*_*p*_, compare (Heldt et al., 2012). We require here that sufficient proteins per virion need to be available in order to reach the half-maximal virion release rate *K*_*D*_. Furthermore, we introduced a second host factor *HF*_*PP*_ for particle production, with a cell line-specific basal production rate *k*_*H*_*F*__*PP*__:

(18) $$\begin{array}{l} \frac{dHF_{PP}}{dt}=k_{HF_{PP}}-{N_{HF_{PP}}v}_{p}. \end{array}$$

The infectious virions (*V*_*INF*_) are released from the cell and are then able to infect naïve cells with a reinfection rate constant *k*_*re*_ (Equation 19); they furthermore are assumed to degrade with rate constant μ_*V*_, thus

(19) $$\begin{array}{l} \frac{dV_{INF}}{dt}=v_{p}-k_{re}V_{INF}-\mu_{V}V_{INF}. \end{array}$$

#### Host Cell Immune Response

We coupled the replication model with a simple model containing the central steps of the cell's IIR to infection. This HIR sub-model (Figure 2) comprises four main processes: (1) The recognition of viral RNA by cellular PRRs, leading to the initiation of a signaling cascade that causes (2) the production and release of IFN. (3) Subsequently, secreted IFN triggers the transcription of mRNAs of hundreds of ISG, which are then (4) translated into ISG proteins that act upon multiple processes in the DV lifecycle. To keep the model simple, we subsume the different ISGs by a single representative species, included in the model by its mRNA (*I*_*R*_) and protein (*I*_*P*_) species.

As mentioned above and to keep the model simple, we include only dsRNA recognition via RIG-I/MDA-5 into the model. As soon as dsRNA is detectable in the cytoplasm, it activates the HIR. We therefore modified the equation for *R*_*R*_ (Equation 14a) as follows (changes in bold), where *k*_*rig*_ describes the rate at which the RIG-I pathway is activated and IFN is produced when cytoplasmic *R*_*R*_ is bound by the receptor:

(14b) $$\begin{array}{l} \frac{dR_{R}}{dt}=\;\mu_{RC}\left(R_{DS}+R_{IDS}\right)-\mu_{R_{V}}R_{R}-k_{rig}R_{R}.\left(14\text{b}\right) \end{array}$$

The dynamics of key components of the HIR, namely intracellular IFN (*F*_*IN*_), secreted IFN (*F*_*EX*_), ISG mRNA (*I*_*R*_), and ISG protein (*I*_*P*_) are given by the following ODEs:

(20) $$\begin{array}{l} \frac{dF_{IN}}{dt}=k_{rig}R_{R}-k_{s}F_{IN}-\mu_{F}F_{IN}, \end{array}$$

(21) $$\begin{array}{l} \frac{dF_{EX}}{dt}=k_{s}F_{IN}-k_{jak}F_{EX}-\mu_{F}F_{EX}, \end{array}$$

(22) $$\begin{array}{l} \frac{dI_{R}}{dt}=k_{jak}F_{ex}-k_{IC}I_{R}\left(Rib{o_{HIR}}_{tot}-IC\right)+k_{t}IC\\ \;-\mu_{I_{R}}I_{R}, \end{array}$$

(23) $$\begin{array}{l} \frac{dIC}{dt}=k_{IC}I_{R}\left(Rib{o_{HIR}}_{tot}-IC\right)-k_{t}IC-\mu_{IC}IC, \end{array}$$

(24) $$\begin{array}{l} \frac{dI_{P}}{dt}=k_{t}IC-\mu_{I_{P}}I_{P}. \end{array}$$

Here, upon recognition of cytoplasmic dsRNA (*R*_*R*_, Equation 14b), the cell produces IFN (*F*_*IN*_, Equation 20) via the RIG-I/MDA-5 pathway with rate constant *k*_*rig*_. IFN either degrades with rate constant μ_*F*_ or is secreted from the cell (*F*_*EX*_, Equation 21) with rate constant *k*_*s*_ and then degrades extracellularly with rate constant μ_*F*_. Extracellularly, *F*_*EX*_ binds to receptors that activate the JAK/STAT pathway, we assume this to happen with rate constant *k*_*jak*_. Activation of the JAK/STAT pathway then triggers the production of ISG mRNAs (*I*_*R*_, Equation 22), which we assume to degrade with rate constant μ_*I*_*R*__. Ribosomes (*Ribo*_*HIR*_) bind to *I*_*R*_ to form a translation initiation complex (*IC*, Equation 23) with rate constant *k*_*IC*_, which in turn degrades with rate constant μ_*IC*_. The subsequent translation of the translation initiation complex (*IC*) with rate constant *k*_*t*_ leads to the production of ISG proteins (*I*_*P*_, Equation 24). We assume that *I*_*P*_ degrades with rate constant μ_*I*_*P*__. Similar to *TC*, *IC* dissociates into *Ribo*_*HIR*_ and *I*_*R*_ (*I*_*R*_ + *Ribo*_*HIR*_ → *IC* → *I*_*P*_ + *I*_*R*_ + *Ribo*_*HIR*_). Note that in order to prevent ribosomal competition in the model, we discriminate between ribosomes used for DV RNA translation (*Ribo*_*DV*_) and ribosomes used for the HIR (*Ribo*_*HIR*_).

The ISG proteins (*I*_*P*_) affect numerous processes in the viral lifecycle. Here we focus on effects it has on the formation of the translation initiation complex (*k*_1_) and the degradation of viral RNA in the cytoplasm (μ_*R*_*V*__) (Diamond, 2014; Castillo Ramirez and Urcuqui-Inchima, 2015). We furthermore assume that the ISGs cannot reach species inside of the replication vesicles, which thus provides a protected environment for viral replication (see Supplementary Material for details). We include these effects into the model by decreasing the corresponding reaction rate constant *k*_1_ to

(25) $$\begin{array}{l} \hat{k_{1}}=\frac{k_{1}}{1+\varepsilon_{k_{1}}I_{P}}, \end{array}$$

and increasing the degradation rate μ_*RV*_ to

(26) $$\begin{array}{l} \hat{\mu_{R_{V}}}=\mu_{R_{V}}\left(1+\varepsilon_{\mu_{RV}}I_{P}\right). \end{array}$$

Furthermore, we take into account that IFN released from infected cells can protect naïve cells from infection by bringing them into an antiviral state, this has been integrated into the model by decreasing the corresponding reaction rate constant *k*_*re*_ to

(27) $$\begin{array}{l} \hat{k_{re}}=\frac{k_{re}}{1+\varepsilon_{k_{re}}F_{EX}}. \end{array}$$

The efficacy constants ε_*_ measures the efficacy of the inhibition on a range from 0 (no effect) to 1 (full inhibition).

#### Viral Countermeasures

DV is not only subjected to the HIR, but viral proteins in turn also impair the host's immune response, thus constituting a negative feedback loop of mutual inhibition. Several viral proteins have been described inhibiting HIR pathway activation. For example, DV NS3, NS4A, and NS5 inhibit the RIG-I pathway activation by the methylation of the DV RNA (DV NS5) or by blocking the RIG-I/MAVS interaction (DV NS4A) (Chazal et al., 2018). Additionally, by promoting the degradation of STAT2, DV NS5 impairs activation of the JAK/STAT pathway and thus ultimately inhibits ISG production upon exposure of the cell to exogenous IFN (Ashour et al., 2009; Mazzon et al., 2009). Therefore, we incorporated the ability of DV to circumvent the HIR in two ways ($\hat{c_{x}}$): (i) by reducing the reaction rate of the RIG-I pathway activation that may lead to a decreased IFN production (*k*_*rig*_), and (ii) by decreasing the reaction rate for the JAK/STAT pathway activation that may result in a decreased ISG expression (*k*_*jak*_). Similarly, to the antiviral HIR effect, we incorporated these viral countermeasure effects into our model

(28) $$\begin{array}{l} \hat{c_{x}}=\frac{c_{x}}{1+\varepsilon_{x}P_{N}}, \end{array}$$

with *c*_*x*_ ∈ {*k*_*rig*_, *k*_*jak*_} and its efficiency constant $\varepsilon_{x}\in \left[10^{-5},1\right]$, dependent on DV non-structural protein concentration. Hence, we replaced the rate constants *k*_*rig*_ and *k*_*jak*_ in equations (14b), (20), (21), and (22) by the terms $\hat{k_{rig}}$ and $\hat{k_{jak}}$ as defined above (see Supplementary Material for details).

### Parameter Estimation

We implemented the mathematical model in Matlab Release 2016b (The Mathworks). Twenty out of the total 56 model parameters were fixed based on evidence from literature, direct calculations or observations made during the optimization process, see Tables S6, S7. In brief, since infection experiments were carried out at a multiplicity of infection (MOI) of 10 and assuming that the fraction of infected cells follows a Poisson distribution (Flint et al., 2009; Wulff et al., 2012), we computed an initial virus concentration of *V*_0_ = 10 infectious virus particles per ml per cell. Washing of cells to remove unbound virus was performed thrice after 1 h for a duration of 6 min, we therefore set ω_*t*_ = 1 *h*, ω_*d*_ = 0.1 *h* and assume a washing strength of ω_*s*_ = 100. Model parameters for translation and replication rates were estimated based on the DV genome length of approximately 10,700 nucleotides and DV polyprotein length of 3,400 amino acids. During the fitting process, we observed no difference whether the ribosomes bind to viral RNA (*R*_*V*_) or host cell mRNA (*I*_*R*_) and set *k*_1_ = *k*_*IC*_. Assuming a translation velocity of 3 to 8 AA/s per polysome and assuming 10 ribosomes bound to each 2,000 AA (Dahari et al., 2009), we obtain $k_{2}=100\;h^{-1}$. The translation rate (*k*_*t*_) for the ISG proteins (*I*_*P*_) was calculated accordingly as $k_{t}=120\;h^{-1}$, using the IFIT1 protein as a representative ISG with a length of 478 AA (Safran et al., 2010). RNA synthesis rate constants were calculated as $k_{4m}=k_{4p}=1.01\;h^{-1}$, using a transcription rate of 180 nt per min (Dahari et al., 2009). Degradation rates for extracellular virus and IFN were set to $\mu_{V}=0.4\;h^{-1}$ and $\mu_{F}=0.15\;h^{-1}$ (Schmid et al., 2015). Note that for simplicity, we assumed that the intracellular IFN degradation equals the extracellular degradation, μ_*F*_. We observed a higher stability of virus within endosomes (μ_*V*_*E*__) compared to extracellular virus (μ_*V*_) and fixed the degradation rate of virus within endosomes to μ_*V*_*E*__ = 0.5·μ_*V*_. The degradation rate for luciferase $\mu_{L}=0.35\;h^{-1}$ as well as the polyprotein cleavage rate $k_{c}=1\;h^{-1}$ were taken from our HCV replication model (Binder et al., 2013). The translation initial complexes *TC* and *IC* are assumed to be more stable than free cytosolic DV RNA genome (*R*_*V*_) and ISG mRNA (*I*_*R*_) due to the bound ribosomes. Therefore, the degradation rates μ_*TC*_ and μ_*IC*_ are assumed to be lower than the degradation rates μ_*R*_*V*__ and μ_*I*_*R*__. The degradation rate for ISG proteins was fixed to $\mu_{I_{P}}=0.03\;h^{-1}$, corresponding to a half-life of *t*_1/2_ = 24 *h* based on literature showing half-lives for ISG proteins in the range of 12 h and 2.3 days (Ronni et al., 1993; Martensen and Justesen, 2004; Haller et al., 2007; Bogunovic et al., 2013). The half-maximal virion release rate (*K*_*D*_) needed for the virus assembly and release term (*v*_*p*_) was approximated based on the experimental measurements of extracellular virus particles. Here, we calculated that in Huh7 cells, *K*_*D*_ = 1.8 virions/ml per measurement time point were produced, while in A549 cells, *K*_*D*_ = 0.7 virions/ml per measurement time point were produced. The number of structural proteins required to produce one virion has been taken from literature with *N*_*P*_*S*__ = 180 molecules/virion (Kuhn et al., 2002). During the fitting process, we observed a 10 times higher basal production rate for the host factor involved in assembly/release in Huh7 cells than in A459 cells. We therefore set $k_{HF_{PP}}^{Huh7}=10\cdot k_{HF_{PP}}^{A549}$. Furthermore, we observed that the initial concentration of the cell line specific host factor involved in virus assembly and release was fitted to the same value and thus set $HF_{PP_{0}}^{Huh7}=HF_{PP_{0}}^{A549}=HF_{PP_{0}}$. However, we assume that the initial host factor (*HF*_*R*_*C*__0__, *HF*_*P*_*P*__0__) and ribosome (*Ribo*_*D*_*V*__0__, *Ribo*_*HI*_*R*__0__) concentrations, as well as the number of consumed host factors in the virus assembly and release process (*N*_*H*_*F*__*PP*__) are ≥1 molecules/virion. The antiviral HIR and DV countermeasure efficiency constants were estimated within $\varepsilon_{*}\in \left[10^{-5},\;1\right]$, while the remaining model parameters have been estimated within the range [10^−5^, 10^3^]. Initial specie concentrations were *V*_*A*_0__ = *V*_*E*_0__ = *V*_*IN*_*F*__0__ = 0 virions/ml for virus species, *R*_*V*_0__ = *TC*_0_ = *R*_*R*_*C*__0__ = *R*_*D*_*S*__0__ = *R*_*ID*_*S*__0__ = *R*_*R*_0__ = *R*_*P*_ = *P*_*P*_0__ = *P*_*S*_0__ = *P*_*N*_0__ = *L*_0_ = *P*_*R*_*C*__0__ = 0 molecules/ml for viral RNA, protein and luciferase species and *F*_*I*_*N*__0__ = *F*_*E*_*X*__0__ = *I*_*R*_0__ = *IC*_0_ = *I*_*P*_0__ = 0 molecules/ml for the IFN and ISG species, while the initial concentrations of host factors (*HF*_*R*_*C*__0__ ≠ *HF*_*P*_*P*__0__ ≠ 0 molecules/ml) and ribosomes (*Ribo*_*D*_*V*__0__ ≠ *Ribo*_*HI*_*R*__0__ ≠ 0 molecules/ml) have been estimated (for more details, see Supplementary Material).

To fit the model to the experimental data, we computed $R_{P}^{tot}$ = (*V_E_* + *R_V_* + *TC* + *R_RC_* + *R_DS_* + *R_IDS_* + *R_R_* + *R_P_*), $V^{tot}=\left(V+V_{A}+V_{INF}\right)$, and $I_{R}^{tot}=\left(I_{R}+IC\right)$ and introduced four scaling factors *fScale*_*L*_, *fScale*_*R*_, *fScale*_*F*_, and *fScale*_*I*_*R*__ to rescale experimental measurements acquired in relative values (Luciferase, DV RNA, and ISG mRNA) and pg/ml (IFN). Remaining free model parameters were then estimated from the experimental data. Parameter estimation was performed using the Data2Dynamics Matlab toolbox (Raue et al., 2015), using a deterministic trust region algorithm (lsqnonlin) with Latin hyper cube multi-start, minimizing the log likelihood function (Raue et al., 2013) (for more details see Supplementary Material). Parameter estimation was performed simultaneously for the Huh7 and A549 cell lines, where only the DV replication sub-model was used in the Huh7 cells and the full model including the immune response sub-model in the A549 cell line. The only other parameters that were allowed to vary between the two cell lines were the initial concentrations of the host factor for the formation of the minus-strand synthesis complex (*HF*_*R*_*C*__0__) as well as the basal production (*k*_*H*_*F*__*PP*__) of the host factor for particle production (*HF*_*P*_*P*__0__). It is likely that more processes are cell line specific, however, here we summarized all model parameters that did not show any impact on the model fit and focused mainly on cell line specific host factor availability and HIR effects. Tables S6, S7 summarize the final, resulting model parameters used after model fitting.

### Model Analysis

#### Simulation of Antiviral Intervention

We used the model to study potential antiviral strategies. For this purpose, we extended the model by effects of direct-acting antiviral drugs (DAAs). A drug efficacy parameter 0 ≤ ε ≤ 1 was introduced to simulate drug effects on viral attachment (*k*_*a*_), viral entry (*k*_*e*_), formation of the translation initiation complex (*k*_1_), translation (*k*_2_), polyprotein cleavage (*k*_*c*_), replication complex formation (*k*_*Pin*_), minus- and (+)RNA synthesis (*k*_4*m*_ and *k*_4*p*_), virus particle production and release (*v*_*p*_), and infection of naïve cells (*k*_*re*_), by multiplying the corresponding parameter with (1 − ε). We then calculated the time-averaged infectious virus particle concentration released from the cell upon drug administration (ε > 0) within a time interval of 5 days (Δ*t* = 120 *h*), normalized to the time-averaged infectious virus concentration without drug treatment (ε = 0) as

(29) $$\begin{array}{l} \psi =\frac{\langle V_{INF}{\left(t\right)}_{T_{\varepsilon >0}}\rangle }{\langle V_{INF}{\left(t\right)}_{T_{\varepsilon =0}}\rangle }, \end{array}$$

with

(30) $$\begin{array}{l} \langle V_{INF}{\left(t\right)}_{T}\rangle =\frac{1}{\Delta t}\int_{t}^{T+\Delta t}dtV_{INF}\left(t\right), \end{array}$$

where *T* refers to the time point of drug administration (*T* ≤ *t* ≤ *T* + Δ*t*).

#### Identifiability and Sensitivity Analysis

We assessed model identifiability using the profile likelihood method, which analyzes both structural and practical identifiability. The profile likelihood method evaluates the change in the likelihood function after modification of one individual model parameter by re-optimizing the remaining model parameters (Raue et al., 2009; Kreutz et al., 2013; Maiwald et al., 2016), thus assessing if changes in a given parameter can be compensated by modifications in other model parameters. Based on the profile likelihood, we calculated 95% confidence intervals on model parameters, which imply parameter identifiability if the confidence interval is finite (Raue et al., 2009, 2015). Local and global sensitivity analysis were carried out in Matlab using the SensSB toolbox (Rodriguez-Fernandez and Banga, 2010) and the extended Fourier Amplitude Sensitivity Test (eFAST) (Marino et al., 2008). Sensitivities with regard to polyprotein (*P*_*P*_), total (+)RNA ($R_{P}^{tot}\;$) and total Virus (*V*^*tot*^) concentrations were calculated for the two time points *t* = 4 *hpi* (early during infection) and *t* = 72 *hpi* (at steady state).

## Results

**Figure 3:**
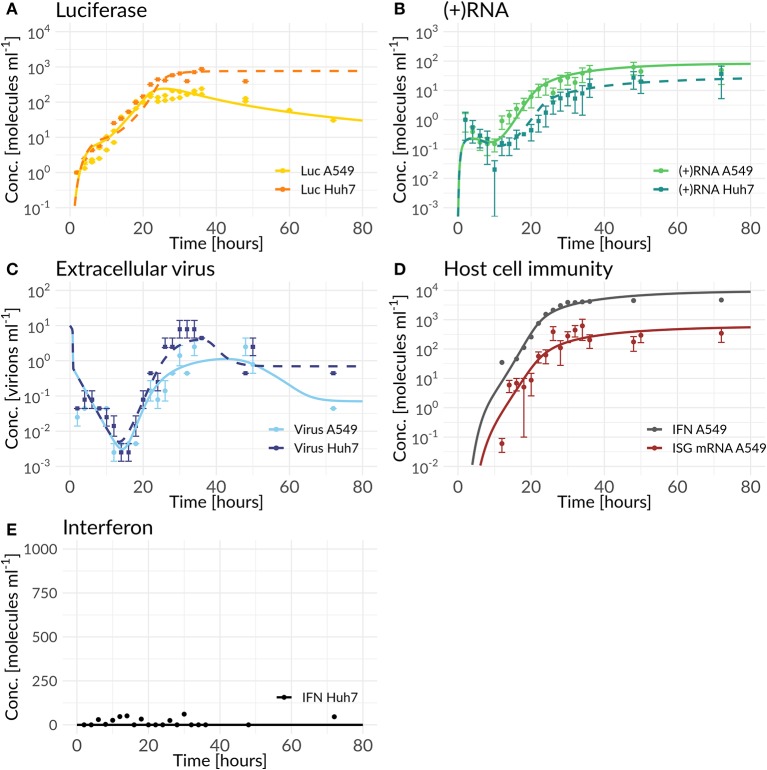
Best model fit of the DV replication model (Huh7) and the coupled model (A549) compared to experimental data measured in Huh7 and A549 cells (for parameter values see Tables S6, S7). Experimental measurements are represented as mean μ± standard deviation σ. The DV replication model and the coupled model were fitted simultaneously to the Huh7 and A549 data sets with cell line specific differences mediated by host factors, the antiviral HIR effect, and DV countermeasures (Equations 1 to 28). **(A)** shows the model fit of luciferase compared to the luciferase measurements (*L* = Luc), **(B)** model fit of total (+)RNA to the (+)RNA measurements ($R_{P}^{tot}$ = (+)RNA), **(C)** model fit of extracellular virus to its measurements (*V*^*tot*^= Virus), **(D)** model fit compared to measurements of the HIR ($I_{R}^{tot}=$ ISG mRNA and *F*_*EX*_ = extracellular IFN), while **(E)** shows the model prediction of the coupled model with cell line specific Huh7 parameter values and the knocked-out RIG-I pathway activation $k_{rig}=0\;h^{-1}$ compared to IFN measured in Huh7 cells.

In order to *in silico* analyze the full DV lifecycle in the absence and presence of HIR mechanisms, we developed a detailed model of the intracellular DV lifecycle and coupled this model to an HIR model, taking into account the antiviral effect of an active immune response on the DV lifecycle as well as DV's ability to in return attenuate the HIR (Figures 1, 2). Model calibration was performed by estimating model parameters simultaneously on experimental data measured in two different cell lines, Huh7 and A549 cells (for details see Materials and Methods). For this purpose, we measured viral polyprotein (luciferase readout), (+)RNA, extracellular virus, and IFN in both cell lines, while ISG mRNA was measured only in A549 cells, as the Huh7 cell do not show activation of the interferon system after DV infection. Polyprotein (luciferase) showed a 1-log_10_ higher translation activity in Huh7 cells compared to A549 cells (Figure 3A). Similarly, Huh7 cells showed a higher extracellular infectious virus concentration compared to A549 cells (Figure 3C). However, in both cell lines, the extracellular virus concentration drops after reaching a peak (~32 hpi in Huh7 and 36 hpi in A549 cells). Nevertheless, against our expectations, DV (+)RNA measurements showed the opposite trend with a faster RNA production in A549 cells (Figure 3B). Additionally, IFN has been measured in both cell lines and showed an increase in secreted IFN in A549 cells (which is followed by ISG mRNA) and a baseline IFN level in Huh7 cells (Figures 3D,E).

Our coupled model, developed based on best biological knowledge, showed high agreement with our experimental cell-line specific measurements after fitting model parameters to the data (Figure 3). Due to the high degree of freedom of the model and in order to prevent overfitting, we analyzed structural and practical identifiability of model parameters. Results are shown in Figure S12; as can be seen in the figure, due to the high model complexity, not all model parameters are identifiable. In particular the parameters for replication within the RC (*k*_3_) and DV RNA export out of the RC (*k*_*Pout*_) are non-identifiable. Both parameters concern the use of newly synthesized plus-strand RNA and reflect the allocation of such newly synthesized RNA to either further rounds of RNA replication (*k*_3_) or to export from the replication compartment and use for protein translation. The fact that these two parameters are non-identifiable is surprising at first, as allocation of newly synthesized RNA between these processes should significantly affect viral replication dynamics. However, this can be explained by other processes that are rate-limiting. In fact, we observed a similar behavior in our HCV replication model (Binder et al., 2013), where in high permissive cell lines the HCV RNA-dependent RNA polymerase became in fact rate limiting for RNA replication inside of the replication vesicles, and led to a similar insensitivity of the model to the parameter *k*_3_. This is reflected in DV as well, with only limited impact of parameters *k*_3_ and *k*_*Pout*_ on the replication dynamics in both cell lines.

### Host Dependency and Restriction Factors

The first question we addressed was cell line specificity. In contrast to our expectations, we observed a faster onset and more efficient viral replication in the HIR-competent A549 cells. Here, our model was not able to describe the DV RNA dynamics in A549 cells that seemed unaffected by the HIR and showed a faster increase and an overall 2.7-fold higher amount of DV RNA (time-averaged concentration) compared to Huh7 cells. Viruses strongly depend on their living hosts and hijack host cell membranes, proteins, and lipids for their own replication. We thus speculated that other host processes explain this difference between the two cell lines. We tested different such potential host factors by including corresponding cell-line specific parameters into the model, compare Table S3, and discriminated between these models using model selection based on Aikaike's Information Criterion (AIC). In line with our previous findings with our published HCV model (Binder et al., 2013), we introduced an unspecific cell line specific host factor, *HF*_*RC*_, that participates in the assembly of the replication complex and RC formation. In model fitting, this host factor showed a higher availability leading to a faster onset of DV replication in A549 cells compared to Huh7 cells (Table S6). We furthermore tested cell-line specific effects on different host factor supported processes such as virus entry, that improved the overall model fit without explaining the cell line specific DV RNA dynamics (see Table S3).

We additionally observed a cell line specific variation in the extracellular DV dynamics, resulting in a 2.8-fold lower extracellular virus concentration (time-averaged) in A549 cells, that could not be described by the HIR alone. Thus, we introduced another unspecific host factor, *HF*_*PP*_, involved in virus particle production and release, with a cell line specific basal production, *k*_*H*_*F*__*PP*__, and a cell line specific virus assembly and release rate, *k*_*p*_. During model parameter estimation, we observed a faster virus assembly and release and an around 10 times faster basal production of the host factor involved in DV assembly and release in Huh7 cells compared to A549 cells. This basal host factor production was the key parameter for the lower virus concentration in A549 cells in steady state. Furthermore, this host factor represented a limiting species for particle production and release, since the drop in the extracellular DV concentration following the peak was associated with a drop in the host factor concentration. Interestingly, the availability of structural proteins had no effect on the drop in released virus (see Figure S1).

### The Antiviral HIR Effect and Viral Countermeasures

During an acute infection, the IIR represents the first line of defense against an invading pathogen. The IIR is mounted by the production of IFN and subsequent ISG translation; the ISGs in turn subsequently inhibit multiple steps in the viral lifecycle. Having developed a detailed model coupling the DV lifecycle with key players of the HIR, we studied the antiviral modes of action in detail and introduced three possible antiviral HIR effects on (i) the translation initiation (*k*_1_), (ii) the degradation of free cytosolic DV RNA (μ_*R*_*V*__), and (iii) the reinfection of naïve cells (k_**re**_) into the model. Selection of these three main mechanisms was based on model selection using the least squared error with the AIC to account for model complexity. For details we refer to the Supplementary Material. By comparing the model fits and its AICs, we observed the best model fit and lowest AIC for a model in which the HIR inhibits the translation initiation complex formation (*k*_1_), followed by a model, that increases the degradation of free cytosolic viral RNA (μ_*R*_*V*__). However, the model considering only the increase in the cytosolic RNA degradation (μ_*R*_*V*__) resulted in a very high cytosolic DV RNA degradation rate of $\hat{\mu_{R_{V}}}=987\;h^{-1}$. The model that led to an antiviral state by inhibiting reinfection of naïve cell infection led to the third best model. Since we are interested in combinatory effects, we chose all three antiviral ISG and IFN dependent effects as our working model.

**Figure 4:**
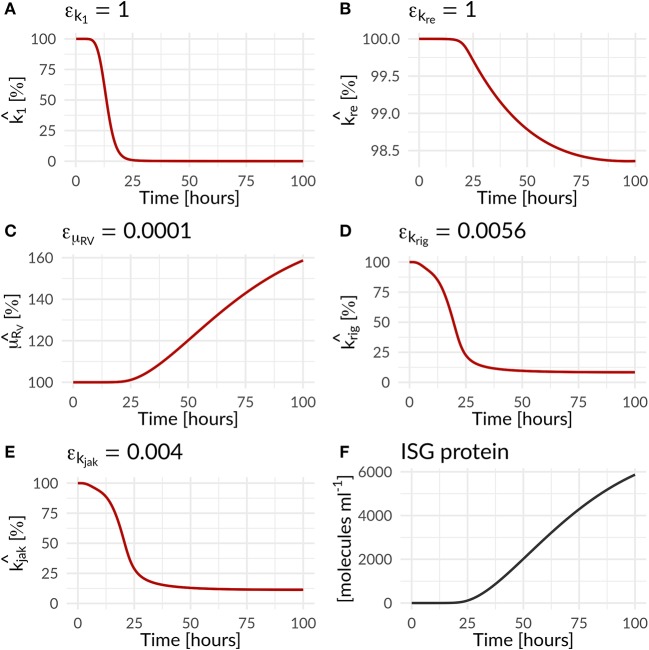
Percentage increase and decrease of model parameters of the antiviral ($\hat{k_{1}},\;\hat{k_{re}},\;\hat{\mu_{R_{V}\;}}$) and countermeasure ($\hat{k_{rig}},\;\hat{k_{jak}}$) effect of the HIR and DV on model parameters as a function of time (Equations 25 to 28; Table 1). The antiviral HIR mediated inhibition of the **(A)** translation initiation complex formation *k*_1_, **(B)** naïve cell infection/reinfection *k*_*re*_, **(C)** cytosolic DV RNA degradation. The DV mediated countermeasure of **(D)** RIG-I pathway *k*_*rig*_ and **(E)** JAK/STAT pathway *k*_*jak*_, as well as **(F)** the ISG protein concentration over time. Note that $\hat{k_{1}}$ and $\hat{\mu_{R_{V}}}$ are ISG dependent, while $\hat{k_{re}}$ is IFN dependent.

In the combined HIR effect model, the inhibition of the translation initiation (*k*_1_) and the reinfection of susceptible cells (*k*_*re*_) by the HIR showed the highest efficacy constants with ε_*k*_1__ = ε_*kre*_ = 1 (Table S7). Comparing the inhibitory effect on the effective rate constants over time, the rate constant for *k*_1_ dropped by 99.983% from its initial value, while *k*_*re*_ showed a negligible 1.6% decrease (Figure 4 and Table 1). The cytoplasmic RNA degradation rate was increased by 58.7%. DV has the ability to evade the HIR by decreasing or inhibiting its own recognition, correspondingly, the rate constants for RIG-I pathway activation and dsRNA recognition was reduced by 93.6%, while the JAK/STAT pathway activation was reduced by 88.6% (Table 1).

**Table 1:** Effect of the immune response on DV replication parameters and of Dengue on parameters of the immune pathways—change in parameter values over 100 h for HIR affected processes in the DV lifecycle and HIR pathways that are targeted by DV: Translation initiation complex formation (*k*_1_), naïve cell infection/reinfection (*k*_*re*_), cytosolic RNA degradation (μ_*RV*_), RIG-I pathway (*k*_*rig*_), and JAK/STAT pathway (*k*_*jak*_).

**Parameter**	**t=0 h**	**t=100 h**	**Unit**	**Increase/Decrease in %**
***k***_**1**_	1,000	0.17	*ml* *molecules*^−1^ *h*^−1^	−99.983%
***k***_***re***_	1e-4	9.8e-5	*h*^−1^	−1.6%
**μ**_***R***_***V***__	2.8	4.4	*h*^−1^	+58.7%
***k***_***rig***_	2.6	0.2	*h*^−1^	−93.6%
***k***_***jak***_	100	11.4	*h*^−1^	−88.6%

**Figure 5:**
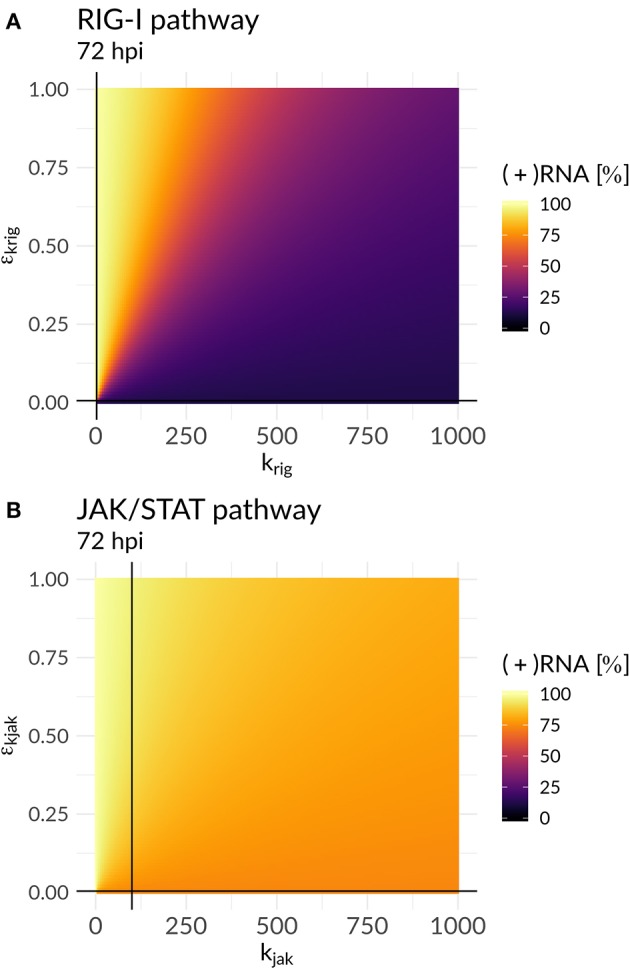
Plus-strand RNA concentration for various model parameter combinations for: **(A)** the antiviral RIG-I pathway activation (*k*_*rig*_) vs. the viral countermeasure targeting the RIG-I pathway (ε_*k*_*rig*__) and **(B)** the JAK/STAT pathway activation (*k*_*jak*_) vs. its viral counteract (ε_*k*_*jak*__). The black lines represent the model parameter combinations that have been estimated from the data (Table S7).

In order to further mathematically analyze the interplay of antiviral effects (*k*_*rig*_ and *k*_*jak*_) and the viral ability to attenuate the HIR (ε_*k*_*rig*__ and ε_*k*_*jak*__), we performed a bifurcation analysis at time 72 hpi. Here, we compared the (+)RNA concentration to various effect combinations: (i) the recognition of DV dsRNA (*k*_*rig*_) vs. DV's ability to attenuate its own recognition (ε_*k*_*rig*__) and (ii) the activation of the JAK/STAT pathway (*k*_*jak*_) vs. DV countermeasures targeting the JAK/STAT pathway (ε_*k*_*jak*__) (Figure 5). In the first scenario, we observed a clear separation: with increasing *k*_*rig*_ the HIR wins and the infection is effectively cleared with only minimal residual (+)RNA, while with increasing ε_*k*_*rig*__ the virus wins and the infection is ongoing. In contrast, in the second scenario we found that increasing the activation of the JAK/STAT signaling pathway (*k*_*jak*_) did not lead to significant decreases in viral RNA levels.

### Antiviral Drug Intervention

**Figure 6:**
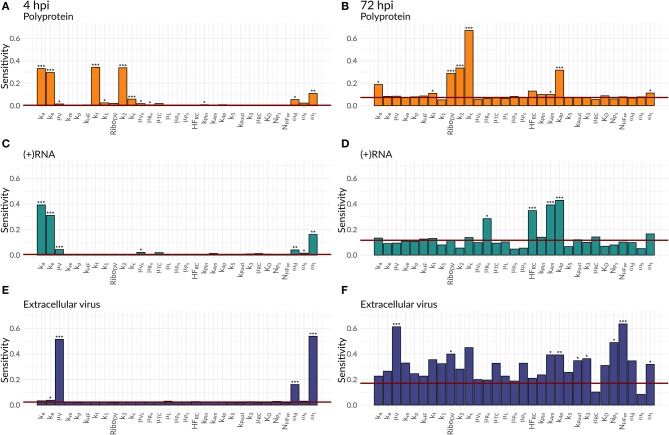
Global Sensitivity analysis performed for the DV replication model for polyprotein **(A,B)**, (+)RNA **(C,D)**, and extracellular virus **(E,F)**, as well as for two different time points: 4 hpi **(A,C,E)** and 72 hpi **(B,D,F)**. The red line is a negative control used for the sensitivity analysis that is not part of the model indicating that sensitivities below the red line are negligible. Significant differences to the negative control have been calculated by performing a *t*-test (*p*-values: *** ≤ 0.001, ** ≤ 0.01, * ≤ 0.05).

**Figure 7:**
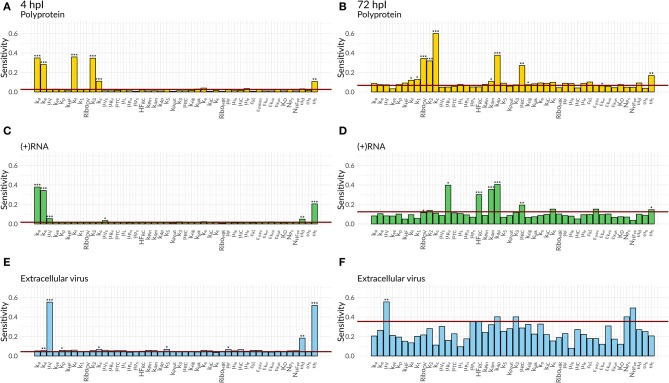
Global Sensitivity analysis performed for the DV replication model coupled to the HIR model for polyprotein **(A,B)**, (+)RNA **(C,D)**, and extracellular virus **(E,F)**, as well as for two different time points: 4 hpi **(A,C,E)** and 72 hpi **(B,D,F)**. The red line is a negative control used for the sensitivity analysis that is not part of the model indicating that sensitivities below the red line are negligible. Significant differences to the negative control have been calculated by performing a *t*-test (*p*-values: *** ≤ 0.001, ** ≤ 0.01, * ≤ 0.05).

We were further interested in using the mathematical model to identify processes with a high impact on the DV lifecycle, as those might constitute attractive targets for antiviral drug development. For this purpose, we performed a global sensitivity analysis to analyze the effect of all model parameters on viral polyprotein, total DV (+)RNA and extracellular virus concentrations at two distinct time points, 4 and 72 hpi (Figures 6, 7).

Both cell lines showed a comparable sensitivity profile for polyprotein, DV (+)RNA, and the extracellular virus and showed high sensitivities to processes associated with cell infection, polyprotein translation and processing, and DV RNA synthesis. For all the three species, early processes in the viral lifecycle were associated with highly significant sensitivities at 4 hpi, such as virus attachment (*k*_*a*_), entry (*k*_*e*_), and fusion (*k*_*f*_), as well as polyprotein translation (*k*_2_). Later in infection (72 hpi), ongoing polyprotein translation as well as processes within the RC dominated in their sensitivities for the three studied species. Especially polyprotein cleavage (*k*_*c*_) became the dominant process with the highest impact of all steps involved in viral protein translation. Of the processes occurring inside of the RC, the most sensitive rate constants were associated with RNA synthesis (*k*_4*m*_, *k*_4*p*_) and the host factor involved in the formation of the RC (*HF*_*RC*_) for both cell lines. For the extracellular virus, the number of host factors (*N*_*H*_*F*__*PP*__) involved in assembly and release showed a higher sensitivity compared to the number of viral structural proteins (*N*_*P*_*S*__). Amongst the HIR model parameters, the RIG-I pathway activation (*k*_*rig*_) showed a slightly higher, significant sensitivity on the polyprotein species. Furthermore, the HIR efficacy constant decreasing the rate constant of the naïve cell infection (ε_μ_*RV*__) showed the highest sensitivity of all antiviral HIR constants, albeit not reaching statistical significance.

**Figure 8:**
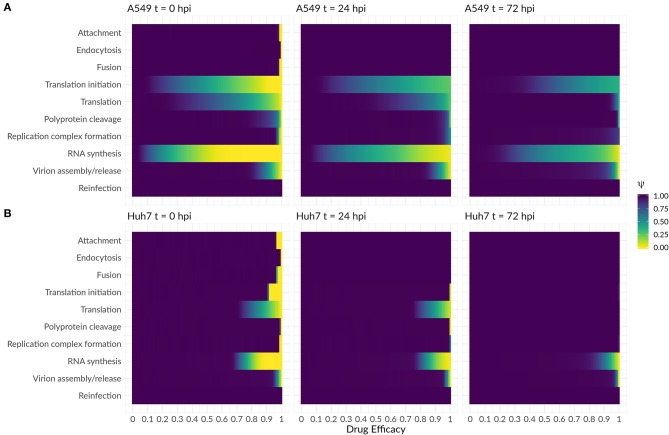
Antiviral intervention study with a drug administration at three different time points (0, 24, and 72 hpi) in **(A)** A549 and **(B)** Huh7 cells. The fold change of infectious virus (ψ) with and without drug administration for the core processes in the DV lifecycle (Equations 29 and 30). A fold change of 1 means no difference between the model with and without drug administration, while a fold change of 0 shows viral eradication to a successful drug treatment.

As a next step, we were interested in the question whether the highly sensitive processes identified in the previous analysis might represent potent drug targets. We therefore performed a theoretical antiviral intervention by simulating a possible drug administration. In this simulation, we monitored the release of infectious virus for 5 days following drug administration. Several processes in the DV lifecycle were inhibited by simulated drug administration at 0 hpi, 24 hpi, and 72 hpi (Figure 8). An early drug administration at 0 hpi led to an efficient viral clearance in both cell lines, using a hypothetical drug acting on any process in the DV lifecycle except for putative drugs targeting reinfection. With the support from the HIR, the overall drug efficacy constants necessary to eradicate the virus in A549 cells were lower. In particular drugs targeting translation initiation and the DV RNA synthesis were able to induce viral clearance even with low drug efficacy constants, and administering a drug targeting the DV RNA synthesis process showed a viral clearance with the lowest drug efficacy constant (ε ≈ 0.5) in A549 cells. For drugs targeting any one of the remaining processes, drug efficacy constants higher than ε ≥ 0.9 were needed to clear the viral infection. Administering a hypothetical drug at 24 and 72 hpi led to comparable viral clearance patterns, but with higher drug efficacy constants. Obviously, if a drug is administered late in the viral lifecycle and targets early processes of the viral lifecycle such as virus attachment, endocytosis and fusion as well as formation of the (membranous) replication compartment, leads to a loss of the drug effect and non-clearance of the DV infection in both cell lines. In both cell lines, the DV infection can still be cleared when blocking DV RNA synthesis and virus assembly/release with <3% DV left with the highest drug efficacy of 1 (thus completely shutting of RNA synthesis and assembly/release), an outcome which cannot be achieved by targeting any of the other processes in the DV lifecycle according to our model simulations.

## Discussion

In the present study, we investigated the intracellular virus replication and HIR dynamics in two different cell lines: Huh7 cells with very low HIR-competence and highly HIR-competent A549 cells. Several cell population models have been developed to analyze DV dynamics under influence of the innate (Schmid et al., 2015) and the adaptive immune response (Ben-Shachar and Koelle, 2014; Clapham et al., 2014, 2016; Ben-Shachar et al., 2016). These models, however, do not take intracellular processes into account and thus lack molecular detail. In order to study the intracellular dynamics during the DV lifecycle, we developed the first mathematical model that reflects the initial dynamics of key components of the intracellular DV genome replication. Our detailed model is derived from previous mathematical models that have been used to describe the intracellular RNA replication of a HCV replicon system after RNA transfection (Dahari et al., 2007; Binder et al., 2013). We extended these models by including virus entry and release of infectious virus particles. Furthermore, we coupled the virus dynamics model to a model of the HIR activation and effector phases in order to study the modes of action of the HIR, and to analyze potential antiviral intervention strategies acting at the level of intracellular mechanisms.

### Host Factors

Our experimental measurements were performed in two different cell lines: Huh7 cells which show no interferon response, and A549 cells showing a strong immune reaction. However, Huh7 cells are based on hepatoma (liver) cells, whereas A549 cells are of pulmonary epithelial origin, thus they likely differ in several other aspects as well. In fact, some characteristics of our experimental data cannot be explained by the lack of an interferon response in the Huh7 cells alone. Contrary to our expectations, we observed a faster onset and more efficient DV genome replication in the immuno-competent A549 cells. We therefore tested which other host factors may explain such cell line specific differences. We set up several different models for such host factors, fitted the corresponding models to the experimental data, and compared different models using AIC; details are given in the Supplementary Material. In our previous HCV study, we have shown that host factors involved in replication complex formation play a crucial role in cell permissiveness and viral replication efficiency (Binder et al., 2013). Similarly, for DV, such a host factor best explained differences in replication efficiency between the two cell lines. According to our model, the more efficient RNA replication (earlier increase and a higher steady state concentration of total (+)RNA in the A549 cell line) is directly associated with a higher concentration of this putative host factor in A549 cells, similar to our previous results considering HCV replication in different Huh7 cell clones (Binder et al., 2013).

Concerning the extracellular virus dynamics, our model was not able to explain the drop in infectious virus titers observed in the experimental data after ~40 h post infection—at different degrees in both cell lines—by a limitation in structural viral proteins. In fact, our simulation results show that structural proteins do not limit the process of particle production and release. Similar to our finding, Heldt et al. (2012) in a mathematical model of influenza A virus replication did not find a limitation in structural proteins and suggested that transport and budding processes might limit the viral production. Furthermore, the drop in virus titers that we observed in our data is qualitatively present in both cell lines, i.e., in the presence and in the absence of the HIR, it is therefore unlikely that it is due to effects of the HIR on virus assembly and release. Therefore, we integrated another unspecific host factor, *HF*_*PP*_, that is involved in virion assembly, maturation, and release into the mathematical model with a cell line-specific basal host factor production and a cell line specific virus assembly and release rate. Fitting of this extended model resulted in a higher production rate of this assembly/release host factor in Huh7 cells, explaining the higher viral steady state level in these cells. Several host factors affecting DV assembly / release are known; we recently employed siRNA screening to identify such factors and described a mechanisms involving Fibroblast Growth Factor Receptor 4 (FGFR4), a host factor supporting DV RNA replication when FGFR4 concentration is high, but leading to increased assembly and maturation of virus particles when this host factor is depleted (Cortese et al., 2019). While FGFR4 is one potential mechanism, the exact identity and mechanisms of host factors differences between A459 and Huh7 based cell lines needs more exploration in the future.

### DV and the HIR

We next employed our mathematical model to characterize the interplay between virus replication on the on hand and the HIR on the other. During DV infection, activation of the interferon system leads to the transcription of hundreds of antiviral ISG proteins at different time points, with effects on multiple steps in the viral lifecycle. In case of HCV, which is closely related to DV and one of the best studied (+)RNA viruses, ISG proteins have been identified to act on almost every step in the HCV replication cycle (Schoggins and Rice, 2011; Metz et al., 2013; Gokhale et al., 2014). Integrating such a multitude of mechanisms into a mathematical model is therefore a daunting task. To keep things simple, we tested different potential antiviral ISG mechanisms individually by including them into our mathematical model and retained the combination of mechanisms leading to the lowest AIC values in model comparison. As we assumed that the intracellular RC protects the newly synthesized viral RNA from detection by and effector mechanisms of the HIR, we did not include any ISG effects on species inside of the RC in our model (Welsch et al., 2009; Belov and van Kuppeveld, 2012; Romero-Brey et al., 2012; Cortese et al., 2017).

According to our single effect models, ISGs inhibiting RNA translation initiation and/or promoting the cytoplasmic RNA degradation led to best fits of the experimental data. However, this model resulted in a 98,600% increase in the degradation rate constant with $\hat{\mu_{R_{V}}}=987\;h^{-1}$, corresponding to an unrealistic RNA half-life of *t*_1/2_ ≈ 2 sec. A model including only IFN dependent inhibition of the reinfection of naïve cells (promoting an antiviral state in susceptible cells) was not able to reproduce the experimental data.

A combination of mechanisms based on model selection criteria described above resulted in a model including ISG effects on (1) translation initiation, (2) cytosolic RNA degradation, and (3) new infection of naïve cells. In this model, DV RNA degradation was increased by 59%, resulting in a degradation rate and half-life of $\hat{\mu_{R_{V}}}=4.4\;h^{-1}$ and *t*_1/2_ = 9 min, respectively. Concerning the reinfection of naïve cells, we observed an inhibition of about 2%, which was rather negligible. Since cells were infected with a high MOI in our experiments, i.e., virtually every cell is infected, viral spread and infection of naïve cells play only a minor role in our experimental data.

While DV is subject to ISG effects, it has also developed several strategies to evade the antiviral HIR by antagonizing and inhibiting the induction of the HIR and the antiviral state induced by it. Several DV NS proteins have been described as highly potent inhibitors of IFN signaling and production. For example, DV NS4B protein has been shown to inhibit STAT1 phosphorylation (Munoz-Jordan et al., 2003), while the DV NS5 protein is well-known to degrade STAT2 and thus result in an inhibition of type I IFN signaling (Ashour et al., 2009). According to our model simulations, during the course of infection, DV inhibits both phases of the HIR, the RLR-mediated induction of IFN by ~94%, as well as IFN signaling through the JAK/STAT signaling pathway by ~ 89%. However, in our model sensitivity analysis at 72 hpi, we found that inhibition of the JAK/STAT signaling pathway may be the more important viral defense mechanism: Increasing the efficiency of the JAK/STAT pathway activation in a sensitivity analysis did not lead to viral eradication, but still resulted in ongoing viral replication with a constant viral RNA concentration of 73%, indicating that DV efficiently counteracts activation of this pathway. In fact, DV's ability to efficiently counteract the JAK/STAT pathway has been confirmed experimentally (Muñoz-Jordán and Fredericksen, 2010). In contrast, we found that increasing the efficiency of DV recognition by the RIG-I pathway led to viral replication at a significantly lower level of only 11% remaining DV RNA. DV's ability to target the JAK/STAT pathway and thus prevent the establishing of an antiviral cellular state mediated by IFN therefore is an efficient and important viral survival mechanism.

### Comparison to HCV

DV and HCV are both (+)RNA viruses of the family *Flaviviridae* and share key features in their lifecycles, but there are striking differences in their clinical manifestation. While a primary dengue infection is acute and occasionally associated with severe complications (DHF, DSS) but does not lead to chronic infection, the rather asymptomatic acute hepatitis C infection may develop into lifelong chronic hepatitis C with life threatening secondary manifestations, such as liver cirrhosis or hepatocellular carcinoma without successful treatment.

Comparing the dynamics of our DV model with our previous HCV model (Binder et al., 2013), we observed that the overall dynamics of luciferase and total (+)RNA in DV is comparable with the HCV dynamics with a highly dynamic initial phase that results in steady states for all the measured species. Most estimated model parameters involved in DV replication showed higher rate constants in DV compared to HCV (Table 2). Considering that DV is causing an acute infection, the faster DV replication seems reasonable, while HCV that may develop into chronicity is in comparison rather slow in its lifecycle.

**Table 2:** Comparison of DV and HCV model parameters.

**Description**	**Parameter**	**DV**	**HCV**
Translation initiation complex formation	***k***_**1**_	1,000 *ml molecules*^−1^ *h*^−1^	1 *molecules*^−1^ *h*^−1^ ^*^
RC formation	***k***_***Pin***_	0.012 *ml*^2^ *molecules*^−2^ *h*^−1^	9.04e-6 *molecules*^−2^ *h*^−1^ ^*^
RNA export	***k***_***Pout***_	1,000 *h*^−1^	0.307 *h*^−1^ ^*^
Further replication within RC	***k***_**3**_	510 *ml molecules*^−1^ *h*^−1^	10^−4^*molecules*^−1^ *h*^−1^ ^*^
Replication intermediate complex formation	***k***_**5**_	1,000 *ml molecules*^−1^ *h*^−1^	10 *molecules*^−1^ *h*^−1^ ^*^
Initial host factor concentration involved in RC formation	***HF***_***RC***_	1 to 4.5 *molecules ml*^−1^	4 to 48 *molecules* ^*^
Initial ribosome concentration	***Ribo***	2.8 *molecules* *ml*^−1^	628 *molecules* ^*^
Viral RNA degradation rate	**μ**_**R**_***V***__	2.8 *h*^−1^	0.363 *h*^−1^
Viral protein degradation rate	**μ**_***P***_	0.001 *to* 0.0025 *h*^−1^	0.06 *h*^−1^

* *Parameter values for HCV have been taken from Binder et al. (2013)*.

### Hypothetical Drug Therapy Against DENV

The recent Zika outbreak in Brazil showed the potential health risks of (re-)emerging viruses. Therefore, a comprehensive understanding of the virus-host interaction is necessary in order to suggest antiviral treatment strategies. According to our global sensitivity analysis and simulated antiviral interventions, the most effective drug targets in the DV lifecycle are processes associated with viral entry, translation, and DV RNA synthesis. However, a drug administration earlier than 24 hpi is highly unrealistic, since dengue symptoms usually start 4 to 7 days following a mosquito bite and last for 3 to 10 days (CDC, 2014). However, targeting viral entry is suggested to prevent viral spread. Later in infection, processes associated with DV RNA synthesis and virus assembly and release still represented the most promising drug targets. The antiviral effect on post-translational and early RNA synthesis proposed by our antiviral drug intervention study might be achievable by drugs like Bromocriptine, which has shown antiviral effects against all DV-Serotypes (Kato et al., 2016). In combination with inhibitors of the DV RNA-dependent RNA polymerase, effective antiviral treatment strategies may be possible. Since all processes in the DV lifecycle depend on host factors, a future antiviral therapy may focus on host factor-targeting with the development of pan-serotype or even pan-viral antiviral drugs. As an example, the global sensitivity analysis of our model showed a high impact of the host factors involved in RC formation on DV RNA and assembly/release. To this end, the inclusion of further host factors in viral replication models might be an important challenge for future, *in-silico* based design of anti-DV treatment strategies.

### Limitations and Outlook

In the current study, we developed the first detailed mathematical model of the intracellular DV lifecycle, coupling viral entry, protein translation, RNA replication and assembly and release with a model of the host cell immune response to infection. It has been shown in literature that stochastic effects play an important role in the activation of the IIR and individual cells in a population respond differently (Rand et al., 2012). Schmid et al. (2015) have shown that on a single cell level the IFN response to DV is stochastic and leaky with a fraction of remaining unprotected cells, in which DV replication is ongoing, emphasizing the complex nature of the IIR and virus-host interactions (Schmid et al., 2015). However, we here studied intracellular processes following DV infection in a single, “average” cell, and thus we do not take into account inter-cell differences. Since cells were infected with a high MOI, where virtually every cell is infected, viral spread is negligible and therefore, the impact of IFN released from infected cells to render non-infected, IFN-exposed cells non-permissive to DV infection is not relevant in our experimental data. We furthermore neglected cell proliferation in our model, which would require a multi-scale model combining effects at the cell population scale with a detailed intracellular model. Overall, we model an average response of an infected cell in order to study the DV lifecycle in absence and presence of the HIR, identifying HIR modes of action and sensitive processes, which might represent suitable targets for antiviral treatment.

In order to keep the HIR sub-model tractable, we simplified the activation of the HIR and took only key players of the HIR into account. Here, we model the recognition of dsRNA that is present in the cytoplasm, assuming that the replication vesicles represent a protective environment in which no RNA recognition occurs. We thus assume that DV replication intermediates are subject of detection, either when leaked into the cytosol through the replication vesicle pore or by replication vesicle decay. However, other cytosolic DV RNA species might be recognized as well, such as highly structured RNA regions in the single-stranded genome.

Furthermore, following the HIR activation, we subsume the different ISG proteins by a single species. This is a simplification that we make to keep the model simple. It is known that different ISGs are active at different time points (Metz et al., 2012), even after uniform IFN treatment (Schmid et al., 2015), hence, we here model an “average” effect. However, with our coupled model, we set the basis to study the DV-host interaction. Modeling the IIR in detail, possibly even coupling it to the adaptive immune response is needed in order to better understand and prevent severe dengue complications and to evaluate treatment strategies that suppress high-level viremia.

## Data Availability Statement

The datasets generated for this study are available on request to the corresponding author.

## Author Contributions

LK, MB, and RB contributed conception and design of the study. BS and AR performed experiments. CZ and LK developed the model. CZ implemented and analyzed the model and data and wrote the first draft of the manuscript. CZ, AR, MB, RB, and LK wrote sections of the manuscript. All authors contributed to manuscript revision, read, and approved the submitted version.

## Conflict of Interest

The authors declare that the research was conducted in the absence of any commercial or financial relationships that could be construed as a potential conflict of interest.

## Abbreviations

AIC: Aikaike's information criterion
(+)RNA: Positive-sense RNA
DAA: Direct acting antiviral
DC: Dendritic cell
DF: Dengue fever
DHF: Dengue haemorrhagic fever
dsRNA: Double-stranded RNA
DSS: Dengue shock syndrome
DV: Dengue virus
rER: Rough Endoplasmic reticulum
HIR: Host cell immune response
HCV: Hepatitis C virus
HF: Host factor
hpi: hours post infection
IFN: Interferon
IIR: Innate immune response
ISG: Interferon stimulated gene
JAK: Janus kinase
MDA-5: Melanoma differentiation associated gene-5
MOI: Multiplicity of infection
NS: Non-structural protein
ODE: Ordinary differential equations
PRR: Pattern recognition receptor
RC: Replication compartment
RIG-I: Retinoic acid inducible gene-I
RLR: RIG-I like receptors
STAT: Signal transducer and activator of transcription factor
TLR: Toll-like receptors
WHO: World health organization.
