# Supplementary material for: A Coupled Mathematical Model of the Intracellular Replication of Dengue Virus and the Host Cell Immune Response to Infection
Source: Front Microbiol. 2020 Apr 29;11:725. doi: 10.3389/fmicb.2020.00725 (PMC7200986; doi:10.3389/fmicb.2020.00725)
Supplement: Supplementary file 1 [file Data_Sheet_1.pdf]

Supplementary Material

A coupled mathematical model of the intracellular replication of dengue virus and the host cell immune response to infection

Carolin Zitzmann[1,2], Bianca Schmid[3,†], Alessia Ruggieri[3], Alan S. Perelson[2], Marco Binder[4], Ralf Bartenschlager[3], Lars Kaderali[1*]

[1] Center for Functional Genomics of Microbes, Institute of Bioinformatics, University Medicine Greifswald, Greifswald, Germany
[2] Theoretical Biology and Biophysics, Los Alamos National Laboratory, Los Alamos, NM 87501, United States
[3] Department of Infectious Diseases, Molecular Virology, Heidelberg University, Heidelberg, Germany
[4] Research Group "Dynamics of Early Viral Infection and the Innate Antiviral Response", Division Virus-Associated Carcinogenesis (F170), German Cancer Research Center (DKFZ), Heidelberg, Germany
[†] present address: CSL Behring GmbH, Philipp-Reis-Str. 2, 65795 Hattersheim, Germany
[*] Correspondence

0. Model implementation details

Model implementation and model fitting have been performed in Matlab Release 2016b (The Mathworks) using the Data2Dynamics toolbox and a deterministic trust region algorithm (lsqnonlin) by minimizing the log-likelihood function [1, 2]:

$$L(\hat{y}|\theta) = \prod_{k=1}^{m} \prod_{i=1}^{d_k} \frac{1}{\sqrt{2\pi\sigma_{ki}^2}} \exp\left(-\frac{1}{2\sigma_{ki}^2}\left(\hat{y}_{ki} - y_k(t_i, \theta)\right)^2\right), \text{ with}$$
$$\hat{L} = -2\log\left(L(\hat{y}|\theta)\right) \qquad (S1)$$

Here, the difference between the observables (y) and the experimental data ($\hat{y}$) are minimized given a set of model parameters (θ). The number of observables is given by m ($k = 1 \dots m$), the number of experimental data by d_k, the measurement time points are given by t_i ($i = 1 \dots d_k$), and the variance of each experimental data point (σ_{ki}^2).

For each model we performed 100 independent optimization runs (final model 1,000 optimization runs) using a Latin hyper cube multi-start approach with 100 different initial parameter values. The advantage of the multi-start approach is a more robust optimization by covering a broad parameter space that leads to a convergence to the global minimum (for more information on the Data2Dynamics toolbox, its algorithms and approaches, see [1, 2]).

Model selection has been performed by comparing the Akaike information criterion (AIC) that is given by

$$AIC = \hat{L} + 2K, \qquad (S2)$$

with the number of model parameters K[3]. Given two model AICs, we preferred the model with an AIC difference less than 2 ($\Delta AIC \leq 2$) [3].

Parameters were estimated simultaneously to the experimental measurements of the Huh7 (plus-strand RNA = R_P^{tot}, luciferase = L, and extracellular virus = V^{tot}) and A549 (plus-strand RNA = R_P^{tot}, luciferase = L, extracellular virus = V^{tot}, extracellular interferon = F_{EX}, and intracellular ISG mRNA = I_R^{tot}) cell lines (for more details see Materials and Methods).

1. The basic model of the dengue virus lifecycle

Our studies of the dengue virus (DV) lifecycle, the antiviral effect of the host cell immune response (HIR) and the DV ability to target the HIR started by studying the DV lifecycle in order to capture the basic dynamics in both cell lines: Huh7 and A549 cells. In the following steps, we extended the model stepwise by integrating cell-line specific differences mediated by the HIR and/or host cell resources and give a comprehensive overview of the model development and selection process.

The basic model of the DV lifecycle coupled to the HIR is given by the set of ODEs (Eqs. 1 to 24) described in the main text (see Materials and Methods). Note that for the basic model we initially neglected host cell resources (host factors) and the effect of/on the HIR, thus the host factor involved in the RC formation ($HF_{RC_0} - R_{RC} = 1$; Eqs. 6, 10, 11, 15, 16) as well as any antiviral HIR effect and DV countermeasures ($\varepsilon_x = 0$; Eqs. 25 to 27).

At first, we studied the drop in extracellular virus that is visible in both cell lines and thus, HIR-independent. For the virus assembly and release process (v_p, Eq. 17; see Materials and Methods), we studied two different functions: a simple and a complex virus assembly and release. First, during the assembly and release process, v_p, newly produced DV RNA (R_P) and DV structural proteins (P_S) are packaged into virions in a simple way with rate k_p, which is given by

$$v_p = k_p R_P P_S. \qquad (S3a)$$

Second, we model DV assembly and release (v_p) more complex using a Michaelis-Menten type equation, as

$$v_p = k_p R_P \frac{P_S}{K_D \cdot N_{P_S} + P_S}, \qquad (S4a)$$

with the number of structural proteins (N_{P_S}) and the half-maximum virion release rate K_D (compare Eq. (17) in Materials and Methods).

Furthermore, we studied components necessary for assembly and release. Additionally to R_P and P_S, a host factor (HF_{PP}) is packaged into the virions whose concentration stays either constant over time

$$\frac{dHF_{PP}}{dt} = 0 \text{ and } HF_{PP} \neq 0. \qquad (S5)$$

or HF_{PP} is produced from the cell with constant rate k_{HF} and the number of HF_{PP} necessary for the assembly and release process ($N_{HF_{PP}}$) are consumed

$$\frac{dHF_{PP}}{dt} = k_{HF_{PP}} - N_{HF_{PP}} v_p \text{ and } HF_{PP} \neq 0. \qquad (S6)$$

The involvement of other species (host factors) in the virus assembly and release process changes Eqs. (S3a) and (S4a) to

$$v_p = k_p R_P \prod_j P_j \qquad (S3b)$$

and

$$v_p = k_p R_P \prod_j \frac{P_j}{K_D \cdot N_{P_j} + P_j}, \qquad (S4b)$$

with $j \in \{P_S, HF_{PP}\}$.

Note that the virus assembly and release rate (k_p), the host factor (HF_{PP}) and its basal production ($k_{HF_{PP}}$) might be cell line specific. However, since 180 structural proteins (N_{P_S}) are consumed during the assembly process [4], we fixed $N_{P_S} = 180$ molecules/virion throughout the model simulations. Additionally, we fixed the following parameter values based on calculations and prior knowledge (DV RNA translation rate $k_2 = 100\ h^{-1}$, ISG mRNA translation rate $k_t = 120\ h^{-1}$, plus- and minus-strand RNA synthesis rate $k_4 = 1.01\ h^{-1}$, initial extracellular virus concentration $V_0 =$ 10 virions/ml/cell, extracellular virus decay $\mu_V = 0.4\ h^{-1}$, interferon decay $\mu_F = 0.15\ h^{-1}$, ISG protein decay $\mu_{I_P} = 0.03\ h^{-1}$; see Materials and Methods for more details).

The models describing the virus assembly and release as a simple reaction or a Michaelis Menten-like function, hence without the involvement of an host factor in the assembly and release process (Model 1A, 1B, 1F, and 1G), did not capture the drop in extracellular virus (Figure S1) suggesting that structural proteins (P_S) are not the species in the assembly and release process determining the extracellular virus dynamics. The models assuming a cell line specific virus assembly and release rate (k_p, Models 1B and 1G) show already a lower AIC and suggest cell line specificity (Table S1). However, we found that a cell line-specific host factor, its basal production and consumption involved in the virion assembly and release process and a cell line specific assembly rate (Model 1J) are necessary to explain the dynamics in the extracellular virus titers in both cell lines which led to the lowest AIC (Figure S2).

Nevertheless, we additionally took the possibility into account, that non-structural proteins (P_N) might be involved in the virus assembly and release process [5] and might represent a limiting species (by neglecting host factors; $j \in \{P_S, P_N\}$ in Eqs. (S3b) and (S4b), Model 1K and 1L). However, these models did not show a limitation in the virus assembly and release process and could not lead to a better model fit.

	Model	p	$\hat{L}$	AIC	ΔAIC
**1A**	Simple virus assembly and release	38	3551.3	3627.3	0
**1B**	Virus assembly and release rate cell line specific	39	3482.4	3560.4	-66.9
**1C**	Host factor with constant concentration	40	3510.1	3590.1	-37.2
**1D**	Host factor with basal production	43	3354.1	3440.1	-187.2
**1E**	Host factor with basal production and cell line specific virus assembly and release rate	44	3248.7	3336.7	-290.6
**1F**	Complex virus assembly and release	40	3620.3	3700.3	+73
**1G**	Virus assembly and release rate cell line specific	41	3457.8	3539.8	-87.5
**1H**	Host factor with constant concentration	43	3513.4	3599.4	-27.9
**1I**	Host factor with basal production	45	3463.4	3553.4	-73.9
**1J**	Host factor with basal production and cell line specific virus assembly and release rate	46	3220.0	**3312.0**	**-315.5**
**1K**	Model 1B and non-structural proteins	40	3530.2	3610.2	-17.1
**1L**	Model 1G and non-structural proteins	42	3451.0	3535.0	-92.3

Table S1: Model fits, number of parameters (p), negative log-likelihood ($\hat{L}$), and AICs for the models without and with a host factor and its basal production for the virus assembly and release process (Eqs. 1 to 24; see Materials and Methods, as well as Eqs. S3 to S6). Models 1A to 1E assume a simple virus and release function (Eq. S3), while models 1F to 1J describe the assembly and release process as a more complex Michaelis Menten-like function (Eq. S4). Models 1K and 1L neglect host cell resources and take the possibility into account, that non-structural proteins might be involved (or limiting) the virus assembly and release process. ΔAIC is showing the difference of the fit of models 1B to 1J to the basic model 1A that served as a reference model. The best model fit with the lowest AIC (in bold) is highlighted in yellow.

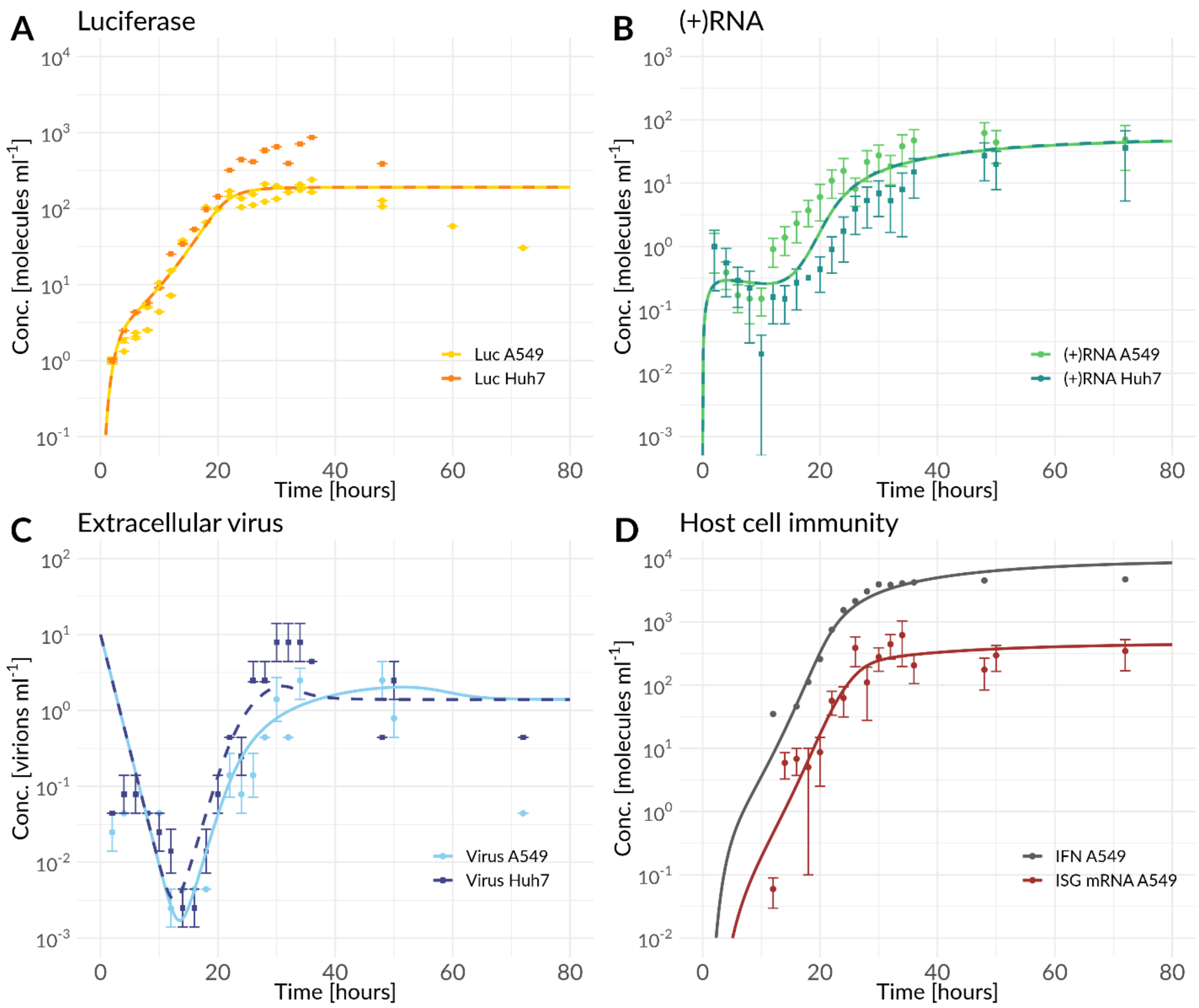

Figure S1: Best model fit amongst the models without host factors required for virus assembly and release (Models 1A, 1B, 1F, and 1G); complex virus assembly and release function with cell line specific virus assembly and release rate (Model 1G). A) shows the model fit of luciferase compared to the luciferase measurements (L = Luc), B) model fit of total (+)RNA to the (+)RNA measurements (R_P^{tot} = (+)RNA), C) model fit of extracellular virus to its measurements (V^{tot}= Virus), D) model fit compared to measurements of the HIR (I_R^{tot}= ISG mRNA and F_{EX} = IFN).

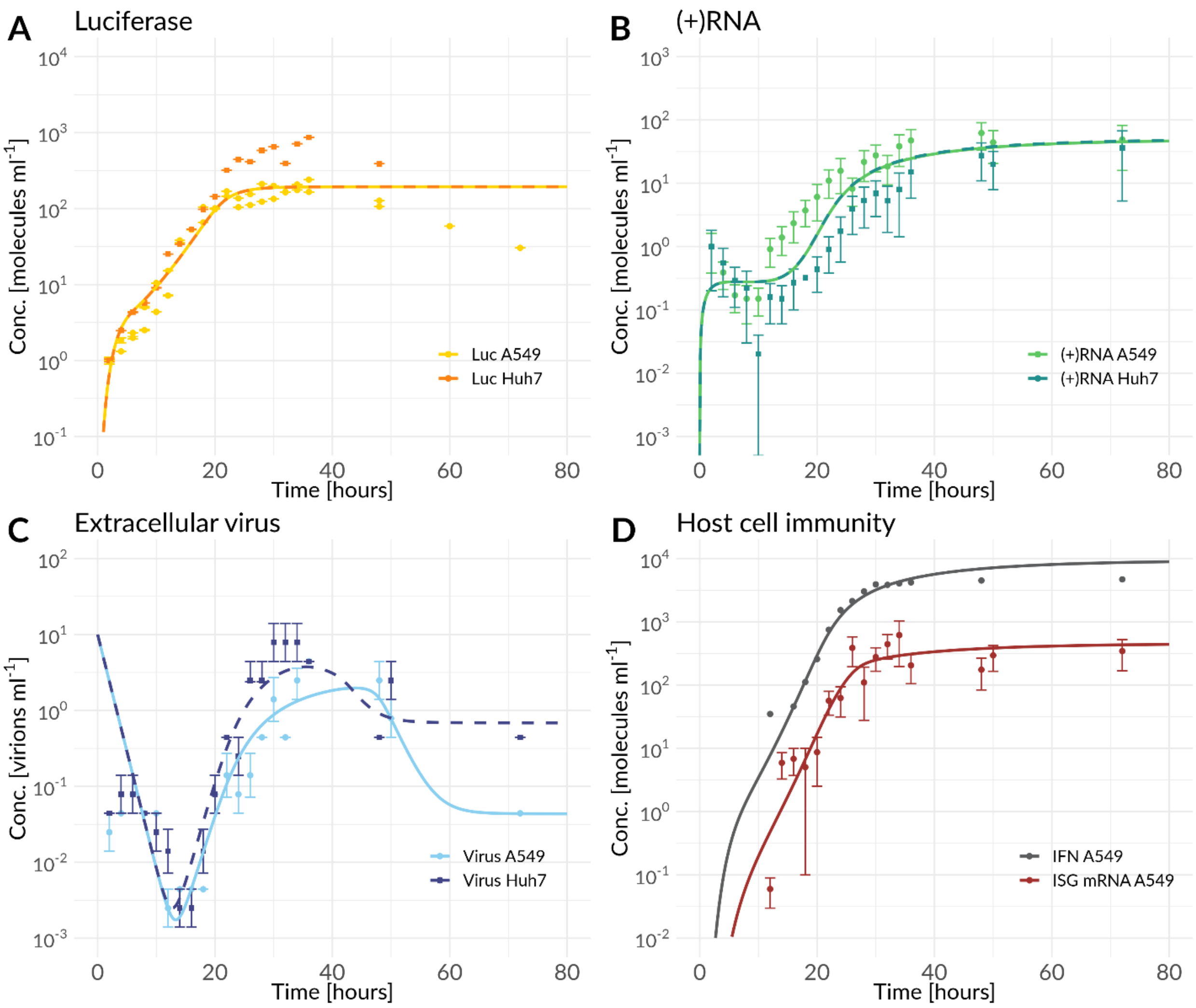

Figure S2: Best model fit with host factors required for virus assembly and release and a host factor basal production rate; complex virus assembly and release function with cell line specific virus assembly and release rate (Model 1J). A) shows the model fit of luciferase compared to the luciferase measurements (L = Luc), B) model fit of total (+)RNA to the (+)RNA measurements (R_P^{tot} = (+)RNA), C) model fit of extracellular virus to its measurements (V^{tot}= Virus), D) model fit compared to measurements of the HIR (I_R^{tot}= ISG mRNA and F_{EX} = IFN).

2. The antiviral effect of the innate immune response on the dengue virus lifecycle

As a next step, we studied the antiviral effect of the HIR on the DV replication, i.e. the decreasing luciferase activity in A549 cells. Note that the basic model does not contain any antiviral effects of the HIR and/or the DV ability to target the HIR. Hence, the basic model, that does only contain the cell line-specific host factor and its basal production for virus assembly and release as well as the cell line specific assembly and release rate (Model 1J), serves as a reference model for model selection and AIC comparison.

The HIR affects the viral lifecycle on multiple steps in the cytoplasm [6, 7] (for more information see main text and references within). Here, we compared several effects of the HIR on: (i) Virus attachment to the cell surface (k_a), (ii) viral cell entry (endocytosis) (k_e), (iii) virus and endosome membrane fusion in order to release the DV genome (k_f), (iv) translation initiation complex formation (k_1), (v) polyprotein cleavage (k_c), (vi) formation of the replication compartment (RC) (k_{Pin}), (vii) virus assembly and release (k_p), (viii) naïve cell infection (k_{re}), (ix) cytosolic virus protein degradation (μ_P), and (x) cytosolic DV RNA degradation (μ_{R_V}). Thus, the process associated reaction rates $k_x \in \{k_a, k_e, k_f, k_1, k_c, k_{Pin}, k_p, k_{re}\}$ are decreased to

$$\widehat{k_x} = \frac{k_x}{1 + \varepsilon_x I_P}, \qquad (S7)$$

and, the degradation rates $\mu_x \in \{\mu_P, \mu_{R_V}\}$ are increased to

$$\widehat{\mu_x} = \mu_x(1 + \varepsilon_x I_P), \qquad (S8)$$

with the HIR efficiency constant $\varepsilon_x \in [10^{-5}, 1]$. Note that all processes are ISG-dependent (I_P) while the naïve cell infection (reinfection, k_{re}) is mediated by extracellular IFN (F_{EX}) and decreases the reaction rate for the reinfection process $p_x \in \{k_{re}\}$ to

$$\widehat{p_x} = \frac{p_x}{1 + \varepsilon_x F_{EX}}, \qquad (S9)$$

(compare Eqs. (25), (26), (27); see Methods and Materials).

The best model was found with the HIR effect on the translation initiation complex formation (k_1, Model 2D) and showed the overall lowest AIC (Table S2, Figure S3), followed by increasing the cytosolic DV RNA degradation rate (μ_{RV}, Model 2I, Figure S4). However, the combination of both HIR effects into one model (Model 2K) did not lead to a lower AIC. Thus, we chose model 2D as our working model for further model extensions, but for every model extension we rechecked combined HIR effects (k_1 and μ_{R_V}).

	Model	Affected parameter	p	$\hat{L}$	AIC	ΔAIC
**1J**	Host factor with basal production and cell line specific virus assembly and release rate		46	3220.0	**3312.0**	**0**
**2A**	(i) HIR effect on virus attachment	k_a	47	3246.8	3340.8	+28.8
**2B**	(ii) HIR effect on endocytosis	k_e	47	3215.0	3309.0	-3.0
**2C**	(iii) HIR effect on fusion	k_f	47	3246.8	3340.8	+28.8
**2D**	(iv) HIR effect on translation initiation complex formation	k_1	47	2938.5	**3033.5**	**-278.5**
**2E**	(v) HIR effect on polyprotein cleavage	k_c	47	3284.2	3378.2	+66.2
**2F**	(vi) HIR effect on RC formation	k_{Pin}	47	3278.0	3372.0	+60.0
**2G**	(vii) HIR effect on virus assembly/release	k_p	47	3260.9	3354.9	+42.9
**2H**	(viii) HIR effect on reinfection	k_{re}	47	3204.7	3298.7	-13.3
**2I**	(xi) HIR effect on RNA degradation in cytoplasm	μ_{RV}	47	3038.0	3132.0	-180.0
**2J**	(x) HIR effect on protein degradation	μ_P	47	3276.8	3370.8	+58.8

Table S2: Model fits, number of parameters (p), negative log-likelihood ($\hat{L}$), and AICs for the models integrating the antiviral effect of the HIR (Models 2B to 2K) compared to the basic model without antiviral HIR effect (Model 1J) (Eqs. 1 to 24, as well as Eqs. S7 to S9; see Materials and Methods, as well as Eqs. S3 to S6). ΔAIC is showing the difference of the fit of models 2A to 2K to the reference model 1J. The best model fit with the lowest AIC (in bold) is highlighted in yellow.

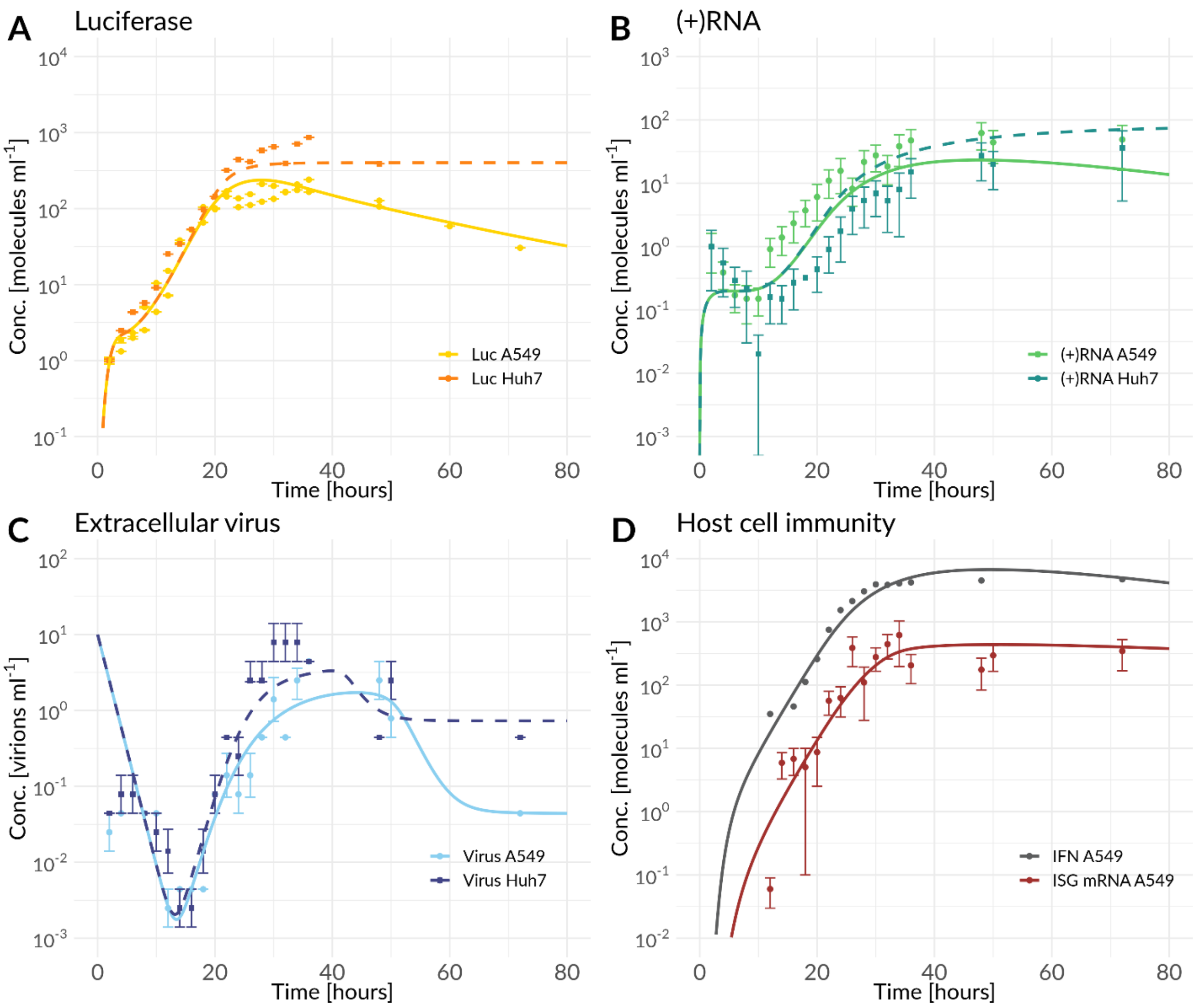

Figure S3: Best model fit with host factors required for virus assembly and release and host factor basal production rate and the HIR effect on the translation initiation complex formation (k_1) (Model 2D). A) shows the model fit of luciferase compared to the luciferase measurements (L = Luc), B) model fit of total (+)RNA to the (+)RNA measurements (R_P^{tot} = (+)RNA), C) model fit of extracellular virus to its measurements (V^{tot}= Virus), D) model fit compared to measurements of the HIR (I_R^{tot}= ISG mRNA and F_{EX} = IFN).

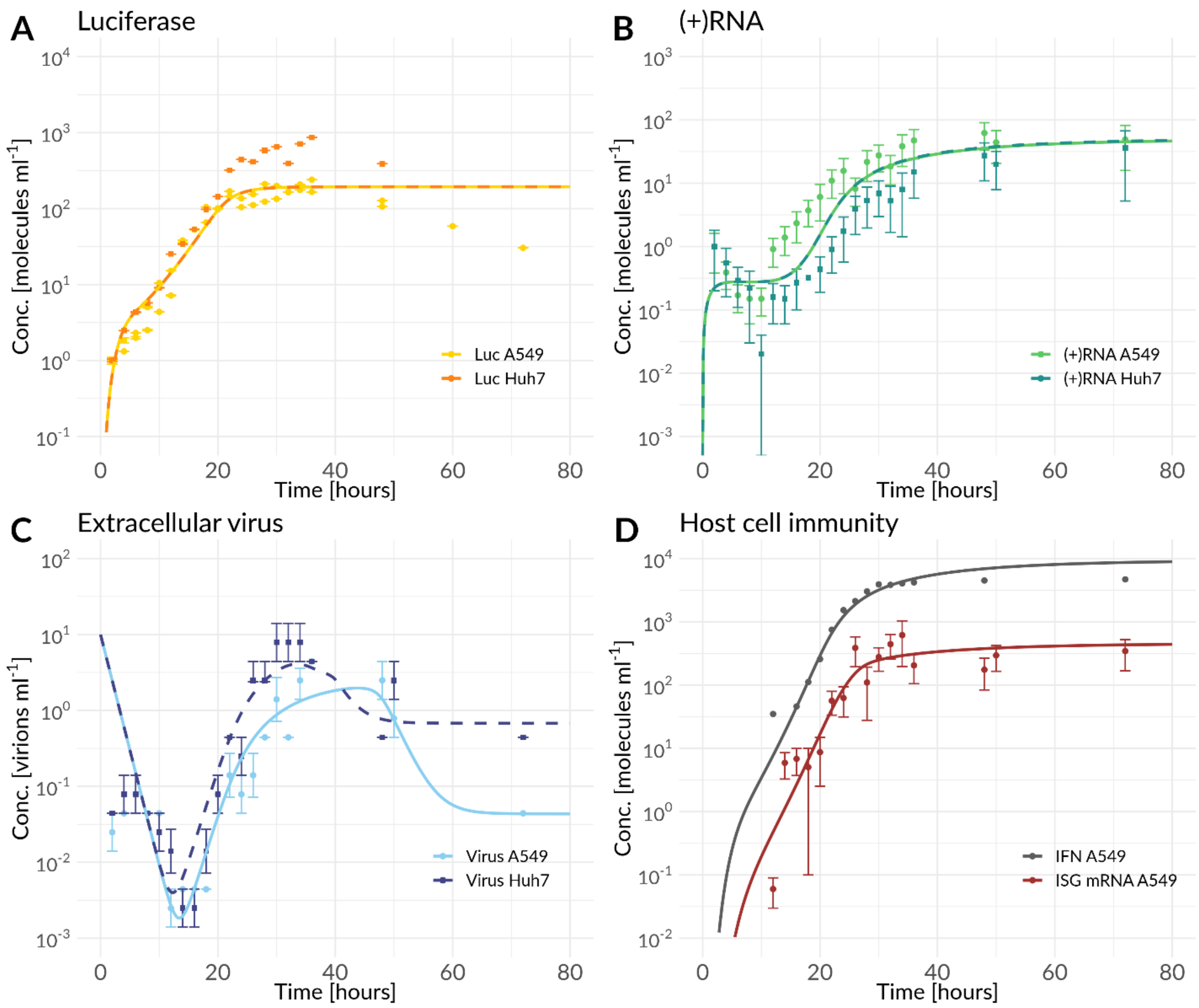

Figure S4: Best model fit with host factors required for virus assembly and release and host factor basal production rate and the HIR effect on the infection of naïve cells (reinfection, k_{re}) (Model 2H). A) shows the model fit of luciferase compared to the luciferase measurements (L = Luc), B) model fit of total (+)RNA to the (+)RNA measurements (R_P^{tot} = (+)RNA), C) model fit of extracellular virus to its measurements (V^{tot}= Virus), D) model fit compared to measurements of the HIR (I_R^{tot}= ISG mRNA and F_{EX} = IFN).

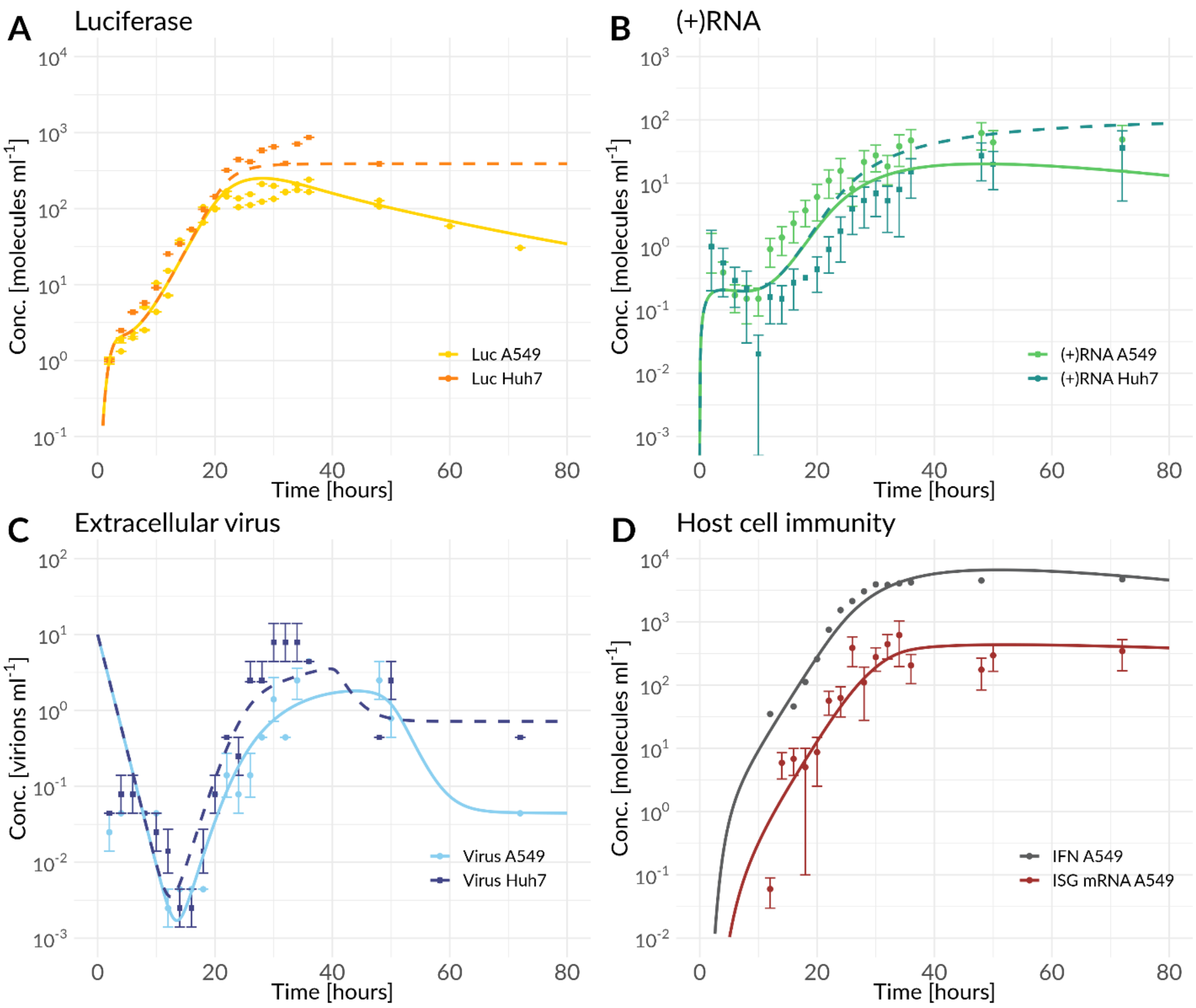

Figure S5: Best model fit with host factors required for virus assembly and release and host factor basal production rate and the HIR effect on the cytosolic DV RNA degradation (μ_{R_V}) (Model 2I). A) shows the model fit of luciferase compared to the luciferase measurements (L = Luc), B) model fit of total (+)RNA to the (+)RNA measurements (R_P^{tot} = (+)RNA), C) model fit of extracellular virus to its measurements (V^{tot}= Virus), D) model fit compared to measurements of the HIR (I_R^{tot}= ISG mRNA and F_{EX} = IFN).

3. Host factors involved in the dengue virus lifecycle

In order to study additional cell line specificities, that might not be explained by the HIR, i.e. the different RNA dynamics in both cell lines, where A549 cells showed a faster RNA production compared to Huh7 cells, we introduced different models, that incorporate host factors for several processes: (i) Virus attachment (k_a, Eq. S10), (ii) virus uptake (endocytosis, k_e, Eq. S11), (iii) fusion of the endosomal and viral membrane to release the DV RNA genome (k_f, Eq. S12), (iv) polyprotein cleavage (k_c, Eq. S13), and (v) formation of the RC (k_{Pin}, Eq. S14). It has been shown, that host factors are involved in most, if not all, processes in the viral lifecycle [8, 9]. Note that a cell line-specific host factor for the virus

packaging and release process is already included in the model. Note furthermore that this model contains antiviral HIR effects in order to study a model that contains cell line specific differences made by the HIR inhibiting and host factors promoting the DV lifecycle.

The model has been extended by multiplying the following terms to the process associated reaction rate:

$$k_a = k_a(HF_A - V_A) \tag{S10}$$

$$k_e = k_e(HF_E - V_E) \tag{S11}$$

$$k_f = k_f(HF_F - R_V) \tag{S12}$$

$$k_c = k_c(HF_C - P_S - P_N) \tag{S13}$$

$$k_{Pin} = k_{Pin}(HF_{RC} - R_{RC}) \text{ and } k_3 = k_3(HF_{RC} - R_{RC}) \tag{S14}$$

Note that the concentrations for every host factor stays constant and thus, $\frac{dHF_i}{dt} = 0$.

By fitting the different host factor models to the data sets of both cell lines, the model that takes into account the possibility of a cell line-specific host factor involved in the entry/endocytosis process showed the overall lowest AIC (Table S3). However, this model was better suited to capture the extracellular virus dynamics, which led to the overall lowest AIC amongst the studied host factor models, but it was not able to describe the DV RNA dynamics in both cell lines (Figure S7). Therefore, according to the AIC, we chose the second best model as our working model with a cell line-specific host factor on the formation of the RC, that was able to describe the extracellular virus and DV RNA dynamics in both cell lines (Figure S8).

Nevertheless, we studied further the model fit with the host factor affected virus entry process, that led to the overall lowest AIC. The model suggests a limitation in the virus entry process, however, the limitation is not cell line specific since in both cell lines the initial host factor concentration was estimated with the same value $HF_E = 9.8\ molecules\ ml^{-1}$. Thus, we introduced a cell washing process, that is in line with the experimental procedure. According to the experimental set-up, the cells were washed to remove unbound virus from the initial infection. This is considered in the model through the term wV in Eq. (1) (see Material and Methods), where w is modelled as

$$w = \omega_s \frac{1}{\sqrt{2\pi\omega_d^2}} \exp\left(-\frac{(t-\omega_t)^2}{2\omega_d^2}\right), \tag{S15}$$

with washing time point ω_t, washing duration ω_d, washing strength ω_s and the independent time variable t. This washing function describes a scaled normal distribution and removes the extracellular virus (V) proportional to ω_s. The values for this function have been chosen based on the experimental condition: the cells were washed after one hour ($\omega_t = 1\ h$) for approximately 6 minutes ($\omega_d = 0.1\ h$). The washing strength is a parameter, that should be high enough to remove the virus and has been set to $\omega_s = 100$ and describes the area under the curve (Figure S6; see for more details [10]).

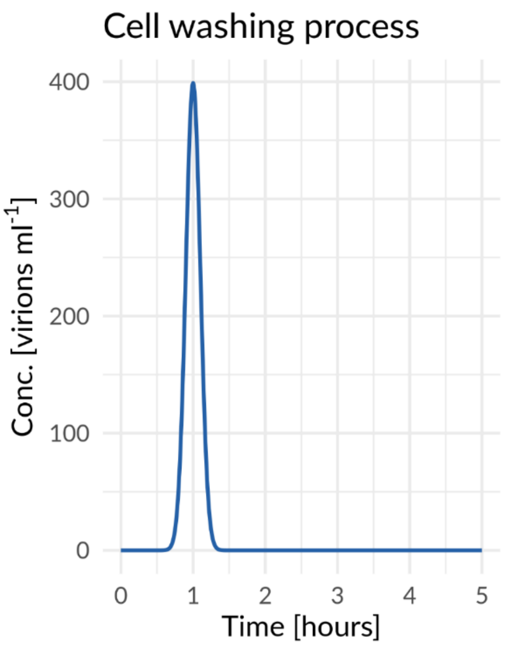

Figure S6: Cell washing process modelled as a scaled normal distribution (Eq. (S15)) with parameter values for washing time point, duration, and strength: $\omega_t = 1\ h$, $\omega_d = 0.1\ h$, and $\omega_s = 100$.

The washing process has been introduced into the model and led to a further improvement of the model fit and a lower AIC (Figure S7, Table S3). Thus, for further model extensions, we chose the model with a cell line-specific host factor involved on the formation of the RC and the modelled cell washing process that is in line with the experimental set-up (Model 3F).

	Model	Affected parameter	p	$\hat{L}$	AIC	ΔAIC
**2D**	HIR effect on translation initiation complex formation	k_1	47	2938.5	3033.5	0
**3A**	(i) HIR effect on k_1 and host factor on virus attachment	k_a	49	2956.3	3054.3	+20.8
**3B**	(ii) HIR effect on k_1 and host factor on endocytosis	k_e	49	1876.7	1974.7	-1058.8
**3C**	(iii) HIR effect on k_1 and host factor on fusion	k_f	49	2901.5	2999.5	-34.0
**3D**	(iv) HIR effect on k_1 and host factor on polyprotein cleavage	k_c	49	2577.5	2675.5	-358.0
**3E**	(v) HIR effect on k_1 and host factor on RC formation	k_{Pin} and k_3	49	2038.7	2136.7	-896.8
**3F**	HIR effect on k_1 and host factor on RC formation, cell washing	k_{Pin}, k_3, and w	52	1569.1	**1673.1**	**-1360.4**

Table S3: Best model fits, number of parameters (p), negative log-likelihood ($\hat{L}$), and AICs for models which have been extended by additional host factors that might explain the faster DV RNA production visible in the A549 cell line measurements (Models 3A to 3E). Model 3F was additionally extended by a cell washing process (Eq. S15). Model 2D serves as a reference model for AIC comparison (ΔAIC). The best model fit with the lowest AIC (in bold) is highlighted in yellow.

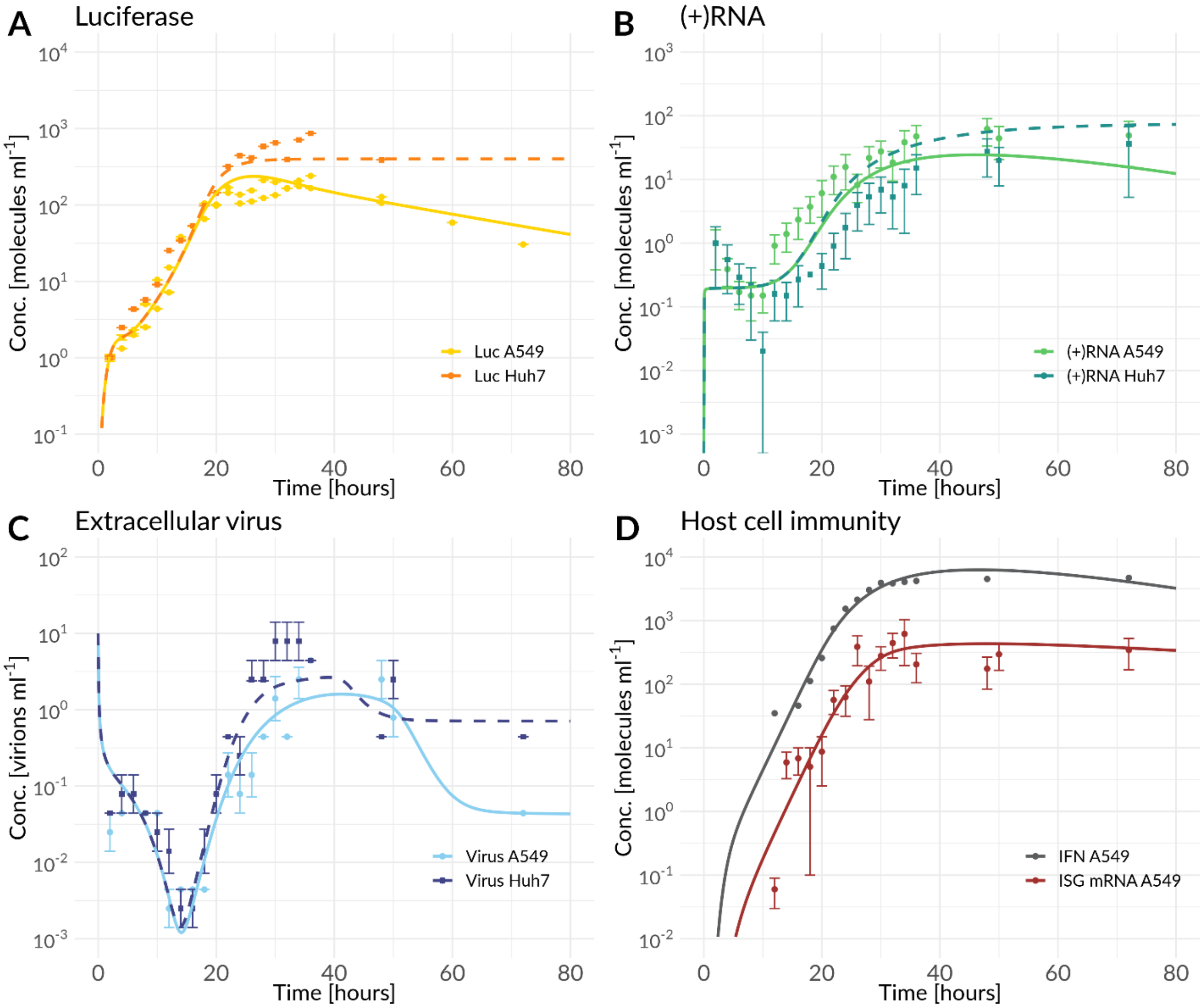

Figure S7: Best fit model with HIR effect on the translation initiation complex formation (k_1) and additional host factors for the endocytosis process (Model 3B). A) shows the model fit of luciferase compared to the luciferase measurements (L = Luc), B) model fit of total (+)RNA to the (+)RNA measurements (R_P^{tot} = (+)RNA), C) model fit of extracellular virus to its measurements (V^{tot}= Virus), D) model fit compared to measurements of the HIR (I_R^{tot}= ISG mRNA and F_{EX} = IFN).

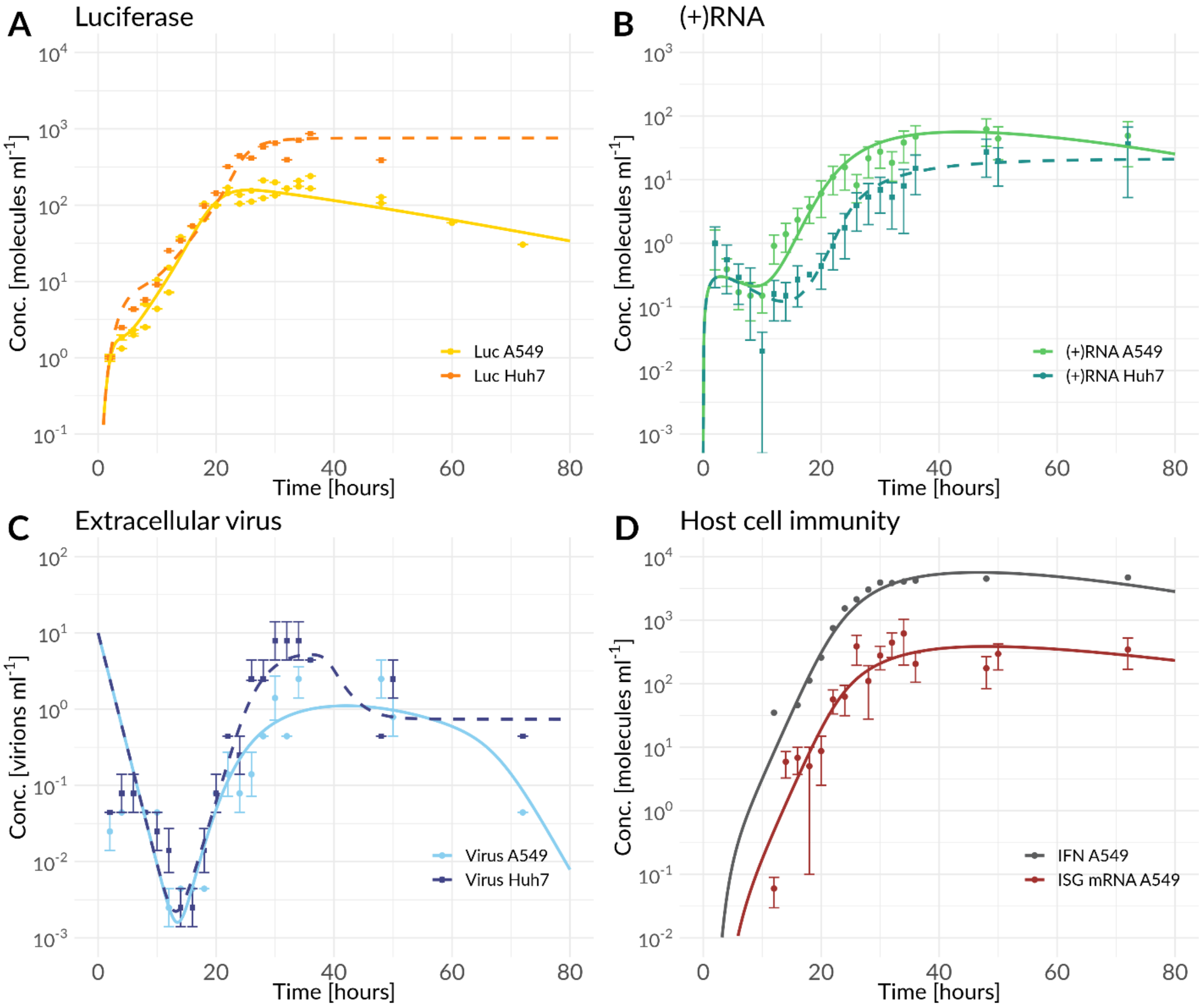

Figure S8: Best fit model with HIR effect on the translation initiation complex formation (k_1) and additional host factors for the RC formation (Model 3E). A) shows the model fit of luciferase compared to the luciferase measurements (L = Luc), B) model fit of total (+)RNA to the (+)RNA measurements (R_P^{tot} = (+)RNA), C) model fit of extracellular virus to its measurements (V^{tot}= Virus), D) model fit compared to measurements of the HIR (I_R^{tot}= ISG mRNA and F_{EX} = IFN).

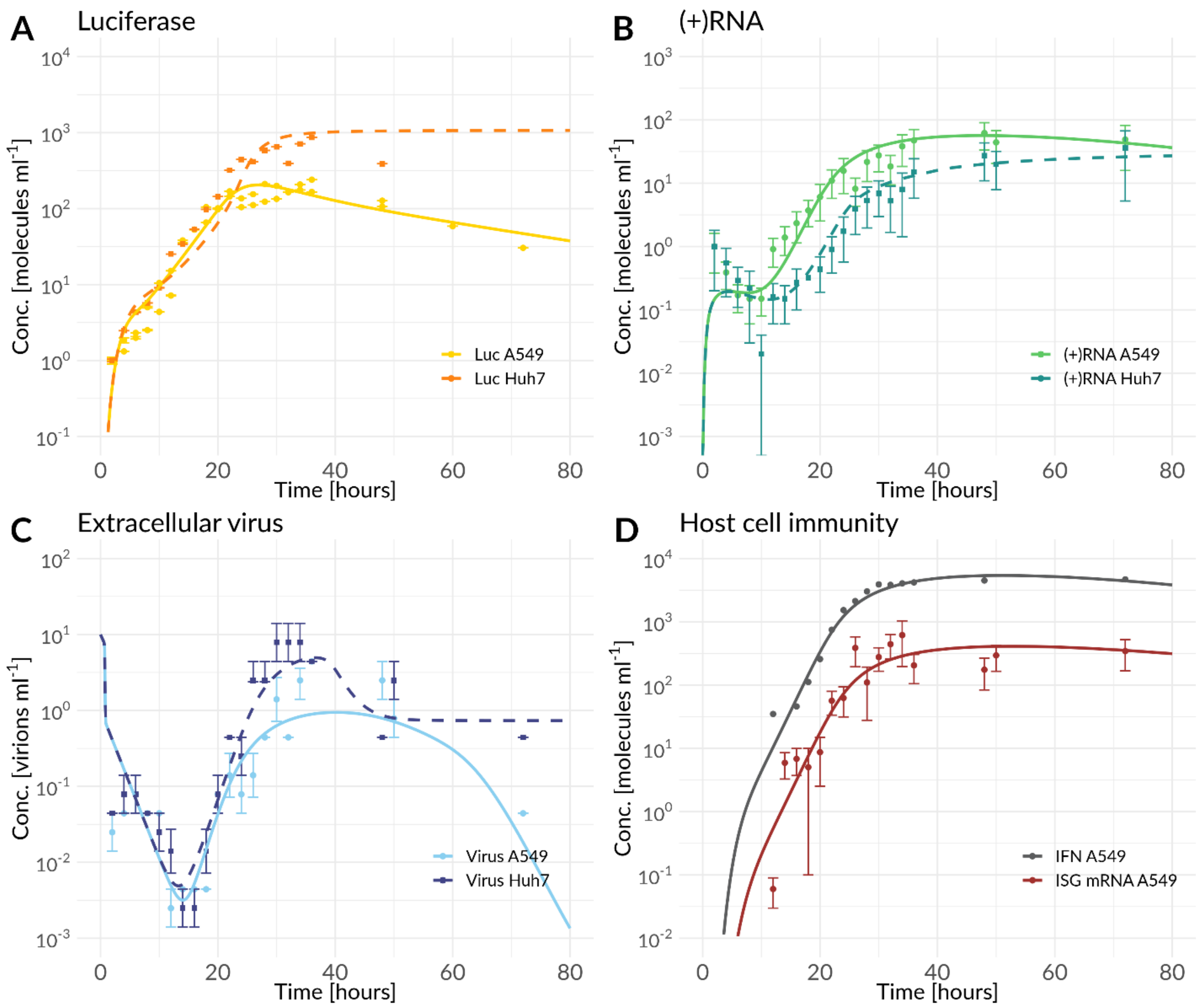

Figure S9: Best fit model with HIR effect on the translation initiation complex formation (k_1) and additional host factors for the RC formation and the integration of a cell washing process (Model 3F). A) shows the model fit of luciferase compared to the luciferase measurements (L = Luc), B) model fit of total (+)RNA to the (+)RNA measurements (R_P^{tot} = (+)RNA), C) model fit of extracellular virus to its measurements (V^{tot}= Virus), D) model fit compared to measurements of the HIR (I_R^{tot}= ISG mRNA and F_{EX} = IFN).

4. The dengue virus countermeasures

Having such a detailed model at hand with antiviral HIR effects and cell-line specificities, we were further interested in the question how DV might target the HIR and studied DV countermeasures. DV developed several mechanisms to target the HIR, especially its own recognition via the RIG-I pathway (k_{rig}) and the IFN signaling of the JAK/STAT pathway (k_{jak}) (for more details see main text and [7, 11–17]). Since the HIR sub-model is highly simplified, we studied both main routes and decreased the

reaction rates of a single pathway or both pathways that are targeted by DV non-structural proteins (P_N) as follows

$$\hat{c_x} = \frac{c_x}{1 + \varepsilon_x P_N}, \tag{S16}$$

with $c_x \in \{k_{rig}, k_{jak}\}$ and the DV countermeasure efficiency $\varepsilon_x \in [10^{-5}, 1]$. Furthermore, the DV countermeasure model was extended by the top three antiviral effects, hence a combination of translation initiation complex formation (k_1), cytosolic DV RNA degradation (μ_{R_V}), and the naïve cell infection (reinfection, k_{re}), which are suggested ISG and IFN antiviral modes of action [18, 19]. The model including the three antiviral HIR effects and two DV countermeasures led to a lower AIC and served as our working model for model complexity reduction (Model 4A, Figure S10, Table S4).

	Model	Affected parameter	p	$\hat{\mathbf{L}}$	AIC	ΔAIC
**3G**	HIR effect on translation initiation complex formation, Host factor on RC formation, cell washing	k_1, k_{Pin} and w	52	1569.1	1673.1	0
**4A**	HIR effect on translation initiation complex formation, cytosolic RNA degradation, and reinfection, Host factor on RC formation, cell washing, DV targeting RIG-I and JAK/STAT pathway	k_{rig} and k_{jak}	56	1522.8	**1634.8**	**-38.3**

Table S4: Best model fit, affected parameters, number of parameters (p), negative log-likelihood ($\hat{L}$), and AICs for the model that take into account the DV countermeasures on the RIG-I (k_{rig}) and/or the JAK/STAT pathway (k_{jak}) by decreasing the corresponding reaction rates (Eq. S16). Model 3G serves as reference models; ΔAIC shows the difference of the DV countermeasure models to the reference model. Additionally, Model 4A was extended by antiviral HIR effect on the translation initiation complex formation (k_1), the cytosolic DV RNA degradation (μ_{R_V}), and the naïve cell infection (reinfection, k_{re}). The best model fit with the lowest AIC (in bold) is highlighted in yellow.

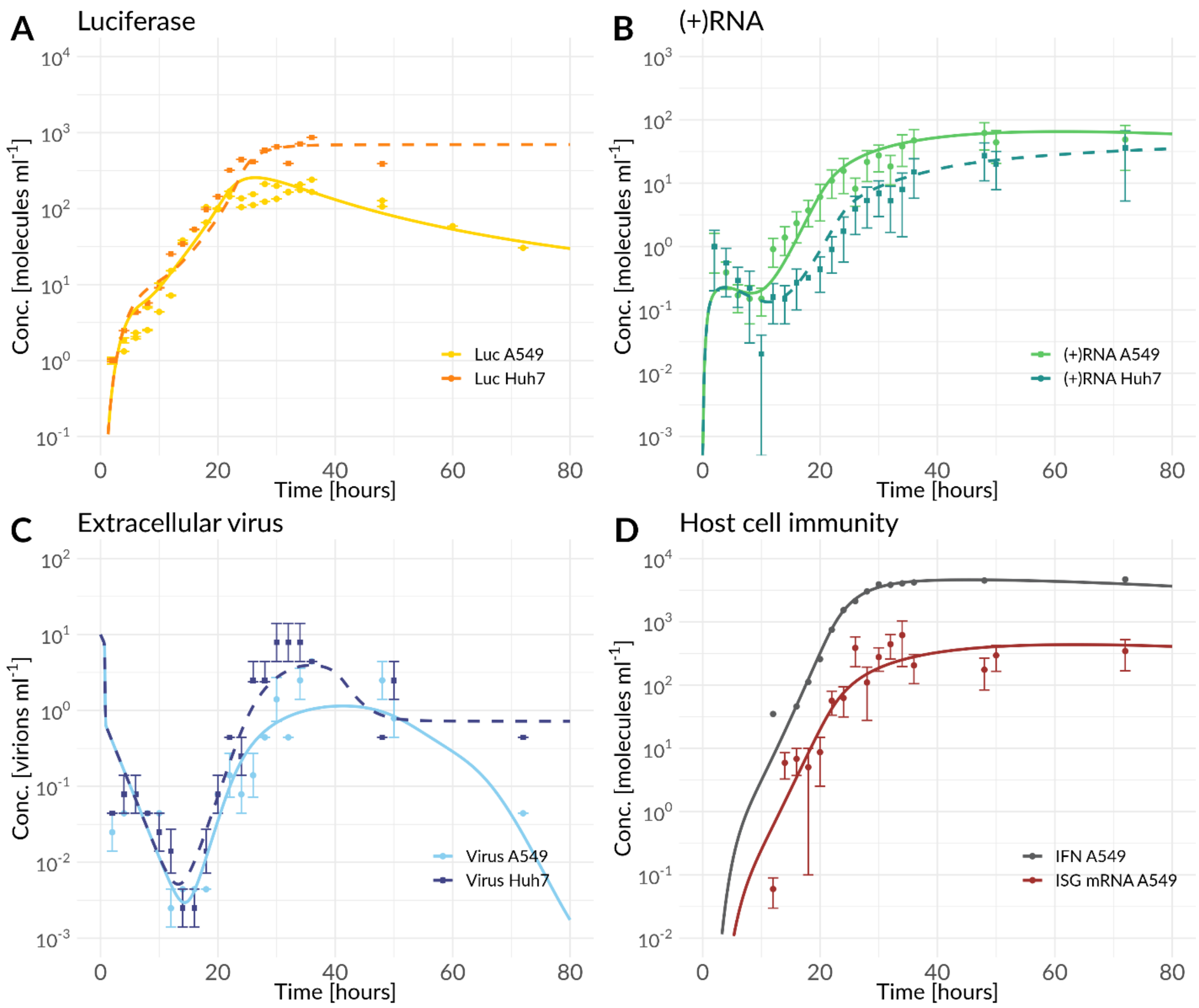

Figure S10: Best fit model with DV countermeasures on the RIG-I and JAK/STAT pathway (Model 4A). The model includes the host factors (and its basal production) for virus assembly and release, host factors for the formation of the RC and the antiviral HIR effects on the translation initiation complex formation and the cytosolic RNA degradation. [Luciferase = L , Plus-strand (+)RNA = R_P^{tot}, and extracellular virus = V^{tot}, extracellular interferon (IFN) = F_{EX}, and ISG mRNA = I_R^{tot}].

5. Model refinement

In order to reduce the model complexity and further improve the model fit, we fixed several parameters based on the following assumptions. Note that based on comparisons of the AICs, we fixed parameters that did either improve the AIC or the difference between the compared two model AICs is less than 2 ($\Delta AIC \leq 2$), which does not prefer one over the other model [3].

(i) The initial concentration for the host factor involved in virus assembly and release were estimated with the same value ($HF_{PP}^{A549} = HF_{PP}^{Huh7} = 58\ molecules\ ml^{-1}$) and thus we set

$HF_{PP}^{A549} = HF_{PP}^{Huh7} = HF_{PP}$. Note that the cell line specificity is mediated from the cell line specific basal production of this host factor ($k_{HF_{PP}}^{A549}$ and $k_{HF_{PP}}^{Huh7}$) and the virus assembly and release rate (k_p^{A549} and k_p^{Huh7}).

(ii) For the basal production of the host factors necessary for virus assembly and release, we observed in Model 1J that the basal host factor production was approximately 10 times faster in Huh7 cells compared to A549 cells. Thus, we assumed $k_{HF_{PP}}^{Huh7} = 10 \cdot k_{HF_{PP}}^{A549}$.

(iii) We observed that virus within endosomes is twice as stable as extracellular virus. With an estimated intracellular virus degradation rate $\mu_{V_E} = 0.2\ h^{-1}$ and a fixed extracellular degradation rate $\mu_V = 0.4\ h^{-1}$based on [12]. Hence, we set $\mu_{V_E} = 0.5 \cdot \mu_V$.

(iv) We assume that there is no difference, whether ribosomes bind to DV RNA or ISG mRNA in order to form translation initiation complexes and since the estimated parameter values for both reaction rates were in the same ($k_1 = k_{IC} = 1000\ ml\ molecule^{-1}h^{-1}$), thus we set $k_1 = k_{IC}$.

(v) The polyprotein cleavage rate was estimated with $k_c = 0.97\ h^{-1}$ and thus we set $k_c = 1\ h^{-1}$ which is in agreement with the polyprotein cleavage rate that has been estimated for HCV in our previous study [20].

(vi) Since the decay rate of the translation initiation complex (μ_{IC}) was estimated higher than the degradation rate of free ISG mRNA (μ_{I_R}), which is biologically not realistic, since the mRNA-ribosome complex might be more stable than free RNA, we introduced the constraint that $\mu_{IC} < \mu_{I_R}$.

By introducing stepwise model assumptions (i) to (vi) into our model, we received our final working model (Table S5, Model 5A, Figure S11) for further analysis. The AIC is 7 points higher than the model, that has not been reduced in its complexity, however, we accept that miner increase of the AIC in order to gain a more robust model. The final model fit and the model identifiability analysis (profile likelihood estimation, see Materials and Methods) are shown in Figures S11 and S12. Model parameter values and 95% confidence intervals are listed in Tables S6 and S7.

	**Model**	**p**	**$\hat{L}$**	**AIC**	**ΔAIC**
**4A**	HIR effect on translation initiation complex formation, cytosolic RNA degradation, and reinfection, Host factor on RC formation, cell washing, DV targeting RIG-I and JAK/STAT pathway	56	1522.8	**1634.8**	0
**5A**	Final model including model reduction steps (i) to (vi)	52	1538.1	**1642.1**	**+7.3**

Table S5: Best model fits, number of parameters (p), negative log-likelihood ($\hat{L}$), and AICs for models that take into account all studied cell line specificities, including host factors, antiviral HIR effects, and DV countermeasures. Model 5A has been reduced in its complexity according to (i) to (vi). Model 4A serves as reference models; ΔAIC shows the difference of the DV countermeasure models to the reference model. The final model and its AIC (in bold) are highlighted in yellow.

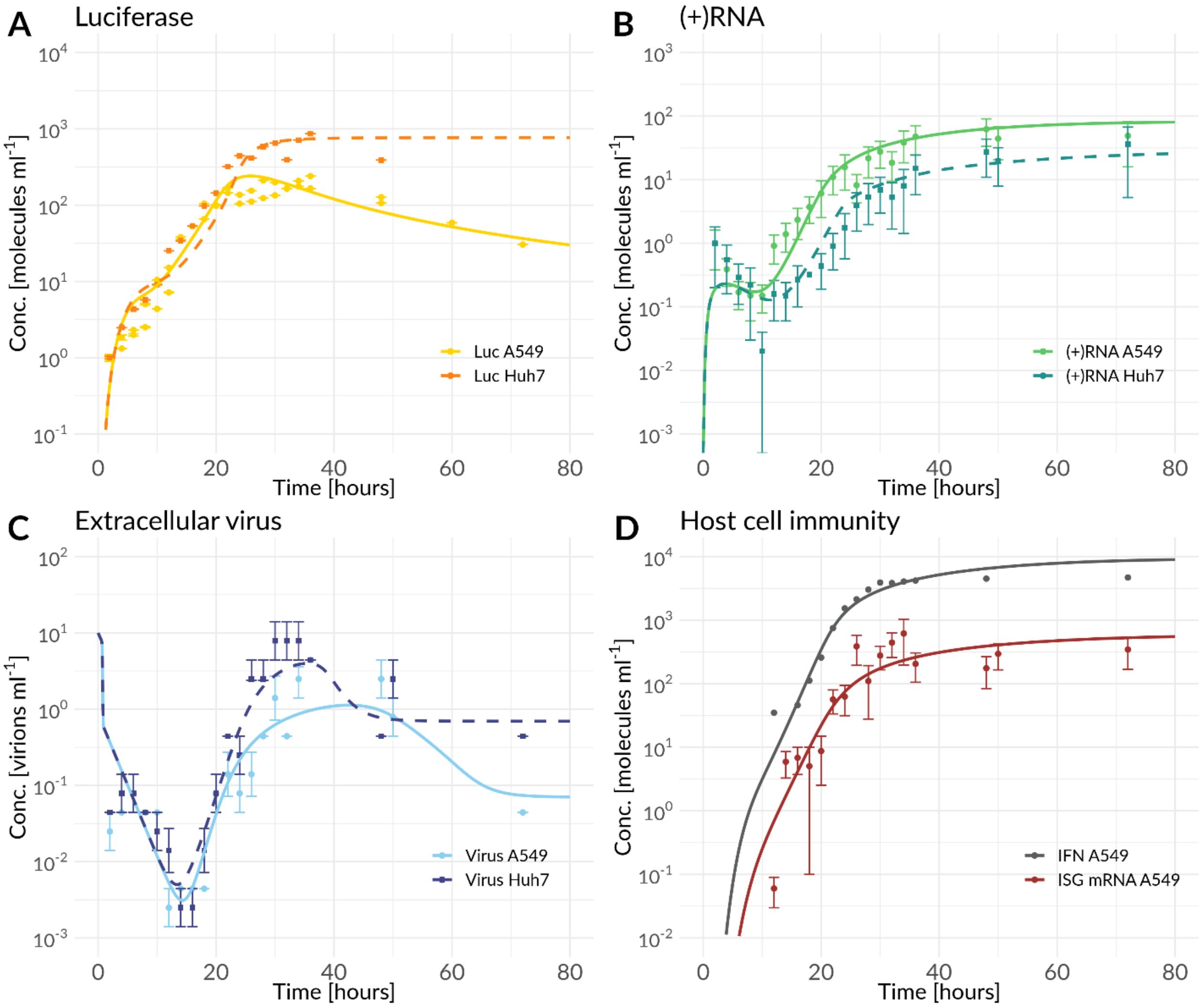

Figure S11: Final fit with the reduced complexity model (Model 5A). A) shows the model fit of luciferase compared to the luciferase measurements (L = Luc), B) model fit of total (+)RNA to the (+)RNA measurements (R_P^{tot} = (+)RNA), C) model fit of extracellular virus to its measurements (V^{tot}= Virus), D) model fit compared to measurements of the HIR (I_R^{tot}= ISG mRNA and F_{EX} = IFN).

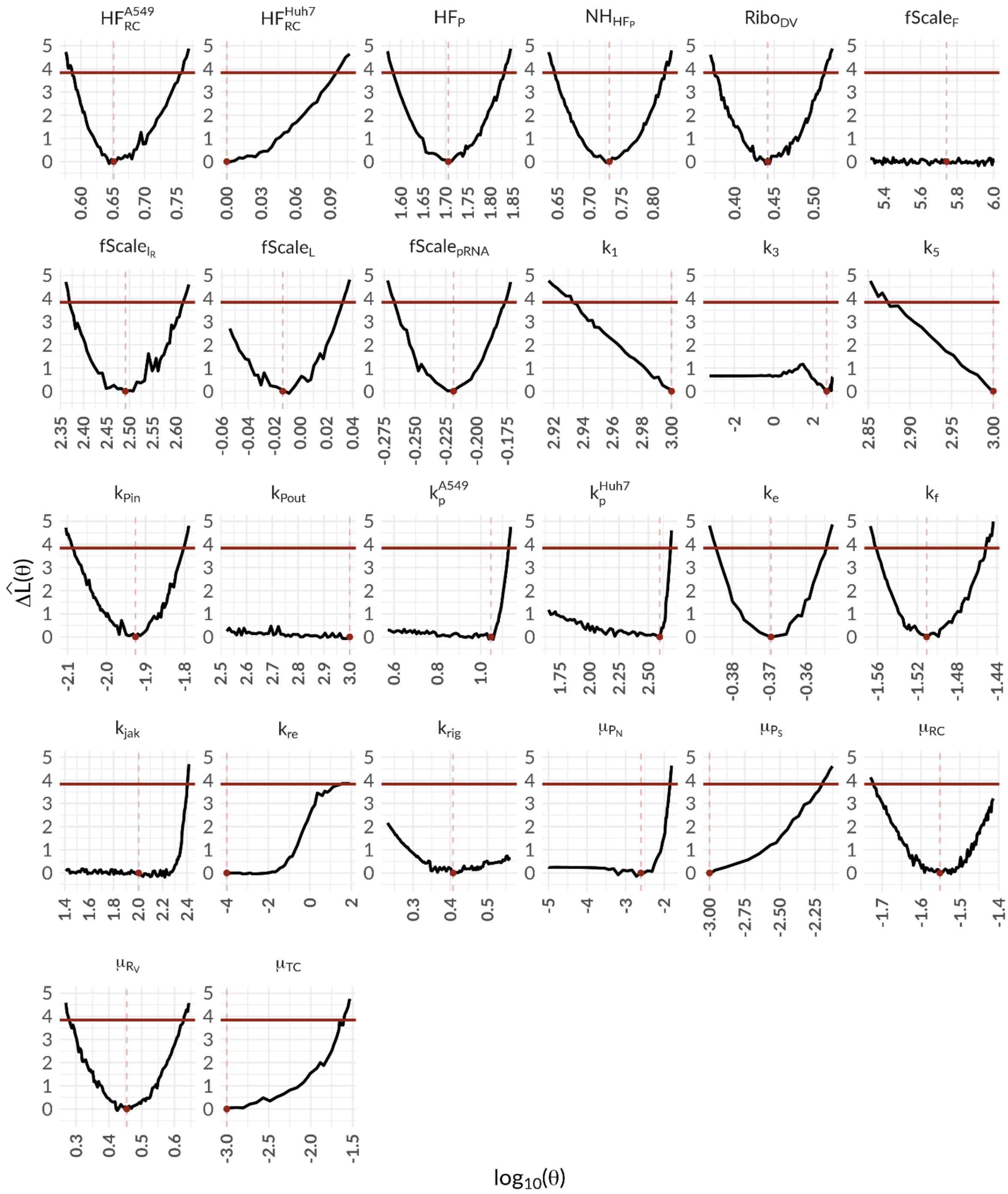

Figure S12: Profile likelihood estimation of estimated model parameters. The red line describes the statistical 95% threshold (95% confidence intervals are listed in Table 1 and 2). A parameter is identifiable if the 95% confidence interval is finite; the black parameter profile line is crossing the statistical threshold. The x-axis shows the scanned parameter profile (as $\log_{10}$ values), y-axis shows the corresponding log-likelihood values ($\Delta\hat{L}(\theta)$ is the difference of the negative log likelihood value). The red dot shows the optimum.

Rate Const.	Definition	Value	Unit	95% CI	Comment
k_a	Attachment rate	0.12	h^{-1}		Fixed after SA/IA
k_e	Endocytosis rate	0.43	h^{-1}	[0.41, 0.44]	
k_f	Fusion rate	0.031	h^{-1}	[0.027, 0.035]	
k_1	Formation rate of translation initiation complex	1000	$ml\ molecules^{-1}h^{-1}$	[857,1000]	
k_2	Translation rate	100 *	h^{-1}		(See Methods)
k_c	Polyprotein cleavage rate	1 *	h^{-1}		(Binder et al., 2013)
k_{Pin}	Formation rate of the plus-strand intermediate complex	0.012	$ml^2\ molecules^{-2}\ h^{-1}$	[0.008, 0.016]	
k_5	Formation rate of the minus-strand intermediate complex	1000	$ml\ molecules^{-1}\ h^{-1}$	[748, 1000]	
k_{4m}	Minus-strand synthesis rate	1.01 *	h^{-1}		(See Methods)
k_{4p}	Plus-strand synthesis rate	1.01 *	h^{-1}		(See Methods)
k_3	Formation rate of the minus-strand intermediate complex	510	$ml\ molecules^{-1}\ h^{-1}$	[0, +∞]	
k_{Pout}	Transport rate out of the RC into the cytoplasm	1000	h^{-1}	[856.5, 1000]	
k_p **Huh7**	Virion release rate in Huh7	11	$ml\ molecules^{-1}h^{-1}$	[0, 486]	
k_p **A549**	Virion release rate in A549	390	$ml\ molecules^{-1}h^{-1}$	[0, 13.4]	
k_{re}	Reinfection rate	0.0001	h^{-1}	[0, 24.4]	
μ_V	Degradation rate of extracellular virus	0.4 *	h^{-1}		(Schmid et al., 2015)
μ_{V_E}	Degradation rate of intracellular virus within endosomes	0.2 *	h^{-1}		(See Methods)
μ_{R_V}	Degradation rate of free cytosolic RNA	2.8	h^{-1}	[1.9, 4.2]	
μ_{TC}	Degradation rate of translation initiation complex	0.001	h^{-1}	[0.001, 0.025]	
μ_{P_N}	Degradation rate of structural proteins	0.0025	h^{-1}	[0, 0.01]	
μ_{P_S}	Degradation rate of non-structural proteins	0.001	h^{-1}	[0.001,0.006]	
μ_L	Degradation rate of luciferase	0.35 *	h^{-1}		(Binder et al., 2013)
μ_{RC}	Degradation rate of species in the replication compartment	0.028	h^{-1}	[0.019, +∞]	
HF_{RC_0} **Huh7**	Initial host factor concentration for k_{Pin} for Huh7	1	$molecules\ ml^{-1}$	[1, 1.24]	
HF_{RC_0} **A549**	Initial host factor concentration for k_{Pin} for A549	4.5	$molecules\ ml^{-1}$	[3.9, 5.7]	
HF_{PP_0}	Initial host factor concentration for k_p for Huh7 and A549	51	$molecules\ ml^{-1}$	[38, 68]	
$k_{HF_{PP}}$ **Huh7**	Basal production rate for HF_{PP} for Huh	1.5 *	$molecules\ ml^{-1}h^{-1}$		(See Methods)
$k_{HF_{PP}}$ **A549**	Basal production rate for HF_{PP} for A549	0.15 *	$molecules\ ml^{-1}h^{-1}$		Fixed after SA/IA
K_D **Huh7**	Half-maximal virion assembly and release rate in Huh7	0.7 *	$virions\ ml^{-1}$		(See Methods)
K_D **A549**	Half-maximal virion assembly and release rate in A549	1.8 *	$virions\ ml^{-1}$		(See Methods)
V_0	Initial virus concentration	10 *	$virions\ ml^{-1}\ cell^{-1}$		(See Methods)
$Ribo_{DV_0}$	Initial ribosome concentration	2.8	$molecules\ ml^{-1}$	[2.4, 3.3]	
N_{P_S}	Number of structural proteins	180 *	$molecules\ virion^{-1}$		(See Methods)
$N_{HF_{PP}}$	Number of host factors	5.4	$molecules\ virion^{-1}$	[4.4, 6.6]	
$fScale_{RNA}$	Scaling factor for RNA	0.6		[0.54, 0.67]	
$fScale_L$	Scaling factor for Luciferase	0.97		[0.88, 1.08]	
ω_d	Washing duration	0.1 *	h		(See Methods)
ω_s	Washing strength	100 *			(See Methods)
ω_t	Washing time point	1 *	h		(See Methods)

Table S6: The table shows model parameters for the virus replication sub-model, estimated simultaneously from data for the Huh7 and A459 cell lines. Parameter values with (*) were fixed based on biological evidence or other considerations (SA = Sensitivity Analysis, IA = Identifiability Analysis).

Rate Constant	Definition	Value	Unit	95% CI	
k_{rig}	RIG-I activation rate	2.6	h^{-1}	[0, +∞]	
k_s	Interferon secretion rate	0.99	h^{-1}		Fixed after SA/IA
k_{jak}	JAK/STAT pathway activation rate	100	h^{-1}	[0, 251]	
k_t	ISG translation rate	120 *	h^{-1}		(See Methods)
μ_F	Degradation rate of intracellular and extracellular interferon	0.15 *	h^{-1}		(Schmid et al., 2015)
μ_{I_R}	Degradation rate of ISG mRNA	1 *	h^{-1}		Fixed after SA/IA
μ_{IC}	Degradation rate of IC	0.1	h^{-1}		(See Methods)
μ_{I_P}	Degradation rate of ISG protein	0.03 *	h^{-1}		(Bogunovic et al., 2013; Haller et al., 2007; Martensen and Justesen, 2004; Ronni et al., 1993)
ε_{k_1}	HIR efficiency constant	1			Fixed after SA/IA
$\varepsilon_{k_{re}}$	HIR efficiency constant	1			Fixed after SA/IA
$\varepsilon_{\mu_{R_V}}$	HIR efficiency constant	0.0001			Fixed after SA/IA
$\varepsilon_{k_{rig}}$	Anti-antiviral efficiency constant	0.0056			Fixed after SA/IA
$\varepsilon_{k_{jak}}$	Anti-antiviral efficiency constant	0.004			Fixed after SA/IA
$Ribo_{HIR_0}$	Initial ribosome concentration used for HIR	100	$molecules\ ml^{-1}$		Fixed after SA/IA
$fScale_{F_{EX}}$	Scaling factor for extracellular interferon	5.5e+5		[0,+∞]	
$fScale_{I_R}$	Scaling factor for ISG mRNA	310		[234, 412]	

Table S7: Model parameters for the HIR sub-model, estimated from data for the A459 cell lines. Parameters value with (*) were fixed based on biological evidence or other considerations (SA = Sensitivity Analysis, IA = Identifiability Analysis).

References

[1] Raue A, Steiert B, Schelker M, et al. Data2Dynamics: A modeling environment tailored to parameter estimation in dynamical systems. *Bioinformatics* 2015; 31: 3558–3560.

[2] Raue A, Schilling M, Bachmann J, et al. Lessons learned from quantitative dynamical modeling in systems biology. *PLoS One* 2013; 8: e74335.

[3] Burnham KP, Anderson DR. Multimodel inference: Understanding AIC and BIC in model selection. *Sociological Methods and Research* 2004; 33: 261–304.

[4] Kuhn RJ, Zhang W, Rossmann MG, et al. Structure of dengue virus: Implications for flavivirus organization, maturation, and fusion. *Cell* 2002; 108: 717–725.

[5] Scaturro P, Cortese M, Chatel-Chaix L, et al. Dengue virus non-structural protein 1 modulates infectious particle production via interaction with the structural proteins. *PLoS Pathog* 2015; 11: e1005277.

[6] Schoggins JW, Rice CM. Interferon-stimulated genes and their antiviral effector functions. *Curr Opin Virol* 2011; 1: 519–525.

[7] Uno N, Ross TM. Dengue virus and the host innate immune response. *Emerg Microbes Infect* 2018; 7: 167.

[8] Neufeldt CJ, Cortese M, Acosta EG, et al. Rewiring cellular networks by members of the

Flaviviridae family. *Nat Rev Microbiol* 2018; 16: 125–142.

[9] Wang Y, Zhang P. Recent advances in the identification of the host factors involved in dengue virus replication. *Virologica Sinica* 2017; 32: 23–31.

[10] Raue A. Data2Dynamics/d2d Wiki/GitHub, https://github.com/Data2Dynamics/d2d/wiki.

[11] Neufeldt CJ, Cortese M, Acosta EG, et al. Rewiring cellular networks by members of the Flaviviridae family. *Nature Reviews Microbiology* 2018; 16: 125–142.

[12] Schmid B, Rinas M, Ruggieri A, et al. Live cell analysis and mathematical modeling identify determinants of attenuation of dengue virus 2'-O-methylation mutant. *PLoS Pathog* 2015; 11: e1005345.

[13] Diamond MS, Pierson TC. Molecular insight into dengue virus pathogenesis and its implications for disease control. *Cell* 2015; 162: 488–492.

[14] Perry ST, Prestwood TR, Lada SM, et al. Cardif-mediated signaling controls the initial innate response to dengue virus in vivo. *J Virol* 2009; 83: 8276–81.

[15] Shresta S, Kyle JL, Snider HM, et al. Interferon-dependent immunity is essential for resistance to primary dengue virus infection in mice, whereas T- and B-cell-dependent immunity are less critical. *J Virol* 2004; 78: 2701–10.

[16] Kao YT, Lai MMC, Yu CY. How dengue virus circumvents innate immunity. *Frontiers in immunology* 2018; 9: 2860.

[17] Ngono AE, Shresta S. Immune response to dengue and zika. *Annu Rev Immunol*; 36. Epub ahead of print 26 April 2018. DOI: 10.1146/annurev-immunol-042617-053142.

[18] Castillo Ramirez JA, Urcuqui-Inchima S. Dengue virus control of type I IFN responses: A history of manipulation and control. *J Interf Cytokine Res* 2015; 35: 421–430.

[19] Diamond MS. IFIT1: A dual sensor and effector molecule that detects non-2′-O methylated viral RNA and inhibits its translation. *Cytokine Growth Factor Rev* 2014; 25: 543–550.

[20] Binder M, Sulaimanov N, Clausznitzer D, et al. Replication vesicles are load- and choke-points in the hepatitis C virus lifecycle. *PLoS Pathog* 2013; 9: e1003561.

[21] Aunins TR, Marsh KA, Subramanya G, et al. Intracellular hepatitis C modeling predicts infection dynamics and viral protein mechanisms. *J Virol* 2018; 92: JVI.02098-17.

[22] Martensen PM, Justesen J. Small ISGs coming forward. *J Interferon Cytokine Res* 2004; 24(1):1-19

[23] Bogunovic D, Boisson-Dupuis S, Casanova J-L. ISG15: leading a double life as a secreted molecule. *Exp Mol Med* 2013; 45: e18.

[24] Haller O, Staeheli P, Kochs G. Interferon-induced Mx proteins in antiviral host defense. *Biochimie* 2007; 89: 812–818.

[25] Ronni T, Melén K, Malygin A, et al. Control of IFN-inducible MxA gene expression in human cells. *J Immunol* 1993; 150: 1715–26.
